# *Pc*-AIF1 Is Expressed in Hemocyte-Rich and Neural Tissues and Links Immune Response and Regeneration in the Snail Model *Pomacea canaliculata*

**DOI:** 10.3390/ijms26189022

**Published:** 2025-09-16

**Authors:** Anita Ferri, Sandro Sacchi, Nicola Franchi, Umberto Rosani, Davide Malagoli

**Affiliations:** 1 Department of Life Sciences, University of Modena and Reggio Emilia, via Giuseppe Campi 213/D, 41125 Modena, Italy; anitaferri@unimore.it (A.F.); sandro.sacchi@unimore.it (S.S.); nicola.franchi@unimore.it (N.F.); 2 Department of Biology, University of Padova, via U. Bassi, 58/B, 35121 Padova, Italy; umberto.rosani@unipd.it; 3 NBFC—National Biodiversity Future Center, 90133 Palermo, Italy

**Keywords:** mollusks, gastropods, immunity, immunocyte, neuroimmunity

## Abstract

Allograft inflammatory factor-1 (AIF1) is a conserved calcium-binding protein involved in inflammatory and neuro-immune responses and expressed in *Pomacea canaliculata* (*Pc*-AIF1) during cephalic tentacle regeneration. Here, we investigated the expression and distribution of *Pc*-AIF1 in control conditions and during cephalic tentacle regeneration. A transcriptomic analysis of 315 RNA-seq datasets revealed maximal *Pc*-AIF1 expression in circulating hemocytes and hemocyte-rich tissues. *Pc*-AIF1 was also highly expressed in neural ganglia. Fluorescence in situ hybridization (FISH) evidenced *Pc*-AIF1 in circulating hemocytes and in the phagocytic hemocyte aggregates in the posterior kidney. qPCR showed the constitutive expression of *Pc-*AIF1 in cerebral ganglia. FISH experiments showed *Pc*-AIF1-positive cells within the cephalic tentacle blastema at 24 h post-amputation (hpa). Even if the amputation left them untouched, both the ipsilateral and contralateral cerebral ganglia increased *Pc*-AIF1 expression until 48 hpa. Immunocytochemical experiments evidenced positive cells to RCA120 (a microglial marker in mammals) among circulating hemocytes, in the connective tissue surrounding the cerebral ganglia, and within the regenerating tentacles. These findings suggest that *Pc*-AIF1 is a neuro-immune marker constitutively expressed in hemocyte populations and neural tissues; it is associated with the immediate hemocyte response to wounding and the neuro-immune interplay during the regeneration of sensory organs.

## 1. Introduction

Allograft inflammatory factor-1 (AIF1) is a calcium-binding scaffold/adaptor protein commonly associated with inflammatory conditions first identified in activated macrophages of humans and rats [1,2,3,4].

The AIF1 gene is in the MHC class III region on human chromosome 6p21.33, located in proximity to key genes involved in immune and inflammatory processes, including TNF-α, TNF-β, and NF-κB [5,6]. Human AIF1 is a 17-kDa cytosolic protein composed of 147 amino acids and belongs to the EF-hand protein family, characterized by helix-loop-helix motifs that bind calcium ions and actin [6,7,8].

In mammals, AIF1 is expressed in different cell types, including activated macrophages, microglial cells, and dendritic cells, where it plays a critical role in modulating immune responses during inflammation [4,9,10,11]. Functionally, AIF1 is implicated in a wide range of cellular processes, including phagocytosis, membrane ruffling, and F-actin cytoskeletal remodeling [10,12]. Its expression is typically induced by pro-inflammatory cytokines such as IFN-γ, TNF-α, and IL-1β [13,14]. Due to these properties, AIF1 has been proposed as a marker of macrophage activation and is considered a key regulator of immune cell polarization and migration [10,12]. Clinically, elevated AIF1 expression has been associated with numerous pathological states, including rheumatoid arthritis, atherosclerosis, cancer (e.g., breast cancer), kidney disease, metabolic syndrome, and various central nervous system disorders [15,16,17]. Its potential as both a biomarker and therapeutic target has been emphasized in the context of transplant rejection, cardiovascular pathologies, and chronic inflammatory diseases [14]. AIF1 is also constitutively expressed in a subset of microglia in the brain and is upregulated in response to various neuropathological conditions, including traumatic brain injury, ischemic infarctions, gliomas, and autoimmune diseases such as encephalomyelitis and uveitis [18,19]. This factor is widely acknowledged as a specific marker for microglia, enabling the identification of these brain-resident immune cells as distinct from neurons and other types of glial cells [20].

AIF1 is conserved among metazoans, and AIF1 orthologs have been described in invertebrates, suggesting that vertebrate and invertebrate AIF1 have originated from a common ancestral gene [21]. Except for mammals, where the functional roles of AIF1 have been deeply investigated, functional studies are limited and concerned only a few species [22,23,24].

AIF1 expression and its link to innate immune response have been documented in hemocytes of several mollusks, including the Pacific oyster *Magallana gigas* (formerly *Crassostrea gigas*) and the gastropod *Haliotis discus discus* [25,26]. In *M. gigas*, AIF1 expression is induced upon exposure to pathogen-associated molecular patterns (PAMPs), resulting in increased phagocytic activity in granular cells [25]. In *H. discus discus*, inflammatory stimuli primarily increase AIF1 expression in hemocytes, with less upregulation in other tissues [26]. Similarly, in the pearl oyster *Pinctada martensii*, AIF1 is constitutively expressed, with the highest levels found in hemocytes. In this bivalve, AIF1 expression increases in response to immune challenges such as bacterial infection or tissue damage, particularly in gills and hemocytes, with peak expression observed within 12 h, suggesting a role in the early phases of acute inflammatory responses [27]. In vitro programmed gene knock-out experiments in *Biomphalaria glabrata* embryonic hemocytes suggested that AIF1 is involved in hemocyte adhesion to the pathogen [28]. In all mollusks studied, AIF1 is constitutively expressed in multiple tissues, including the mantle, gills, hepatopancreas, and foot, but the highest expression levels are consistently found in hemocytes [21,29]. Additionally, AIF1 expression has been documented during cephalic tentacle regeneration in the freshwater snail *Pomacea canaliculata* [30] and in neuro-immune crosstalk during neural repair in the annelid *Hirudo medicinalis* [21]. This supports the idea that AIF1’s role in responding to inflammatory and neuropathological conditions has ancient evolutionary roots [21] and that, in mollusks, it represents a well-conserved mediator in the early immune response. However, information on the involvement of AIF-1 in the crosstalk between neurons and microglia-like cells during neural repair is unavailable for mollusks. In this respect, *P. canaliculata* probably represents one of the best molluscan models, as it has recently been demonstrated to be a genetically tractable model system for studying the regeneration in adult snails of complex sensorial organs, such as the eye [31] or the cephalic tentacles [30], *P. canaliculata* has recently gained attention also as a model organism for studying the invertebrate innate immune system due to its remarkable adaptability to diverse environmental conditions [32,33,34,35], its role as an intermediate host for the parasitic nematode *Angiostrongylus cantonensis* that affects humans [36]. Furthermore, *P. canaliculata* is an attractive model due to its ease of maintenance and culture, the possibility to follow the development *ex-ovo,* and the availability of genomic, transcriptomic, and proteomic data for specific organs [31,37,38,39,40].

As for the other invertebrates, *P. canaliculata* relies exclusively on innate immunity, which consists of both humoral and cellular components, the latter represented by hemocytes [41,42]. In addition to circulating hemocytes, tissue-resident hemocytes have been also described. While their full range of functions remains to be investigated, connections with immunity, phagocytosis, and regeneration have been observed [30,43]. For example, permanent aggregates of phagocytic hemocyte-like cells have been observed in the posterior kidney (PK) of the snail [43,44]. In addition, hemocytes can leave the circulation and contribute as infiltrating cells to the regeneration of organs that typically contain low numbers of these immune cells. This phenomenon has been documented in the regeneration of the cephalic tentacle in *P. canaliculata*, where an accumulation of hemocytes is observed at the regenerating blastema as early as 12 h post amputation (hpa). In this context, the expression of *Pc*-AIF1 has been demonstrated by qPCR, although the cells producing *Pc*-AIF1 have not been identified yet [45].

Due to the limited information available on the identity of *Pc-*AIF1-expressing cells and their potential involvement in neuro-immune crosstalk, this study investigated *Pc*-AIF1 expression both in control snails and during cephalic tentacle regeneration. Specifically, we analyzed the expression of *Pc*-AIF1 in 315 RNA sequencing (RNA-seq) datasets available for *P. canaliculata*. We also analyzed the expression of *Pc*-AIF1 using fluorescence in situ hybridization (FISH), in hemocytes and in tissues containing either resident phagocytic hemocytes (PK) or infiltrating hemocytes (regenerating tentacle). Additionally, FISH was performed on cerebral ganglia as these structures are directly involved in innervating the sensory organs. As AIF1, also known as Iba-1, is considered a marker of microglial cells in vertebrates [20,46,47], and in the absence of information regarding immune cells associated with neural tissue in *P. canaliculata*, immunohistochemical staining using *Ricinus communis* agglutinin 1 (RCA120), which has already been used to stain microglial cells in mammals and microglia-like cells in the Mediterranean mussel, *Mytilus galloprovincialis* [48,49] was conducted to investigate the possible presence of microglia-like cells in *Pomacea* target organs. Additionally, *Pc*-AIF1 expression was examined by quantitative PCR (RT-qPCR) in the cerebral ganglia after tentacle amputation to evaluate neural response during the regenerative process.

## 2. Results

### 2.1. Evaluation of Pc-AIF1 Expression Levels and Validation

**Figure 1:**
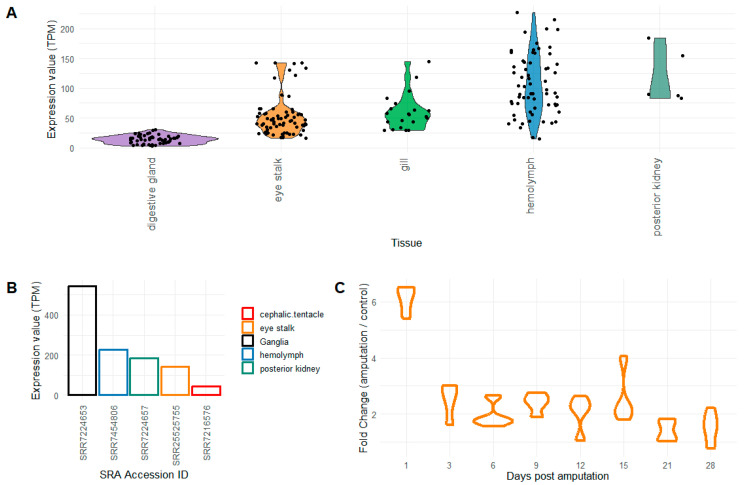
Expression levels of *Pc*-AIF1 are evaluated by whole transcriptome sequencing. (**A**) The boxplot depicted the distribution of the expression levels, reported as transcripts per million (TPM) in a selection of tissues. (**B**) The bar plot indicated the sample with the top *Pc*-AIF1 expression levels among samples of the same origin. (**C**) The boxplot plot indicated the expression fold of *Pc-AIF1* compared to the intact eye stalks along the time-course experiment. All the details related to the expression datasets are reported in the metadata table (Table S1).

Expression data revealed that *Pc*-AIF1 was always detectable in the 315 tested datasets available on NCBI SRA (Figure S1), with hemolymph, gills, and eye stalks possessing the highest average expression levels of *Pc-*AIF1 (LOC112566076, Figure 1A). Although not supported by biological replicates, a unique sample of snail ganglia (SRR7224653) showed the highest expression level in absolute (544 TPMs), followed by hemolymph and heart samples (Figure 1B). Notably, in a time-course experiment designed to study the regeneration process in the eyestalk, *Pc-*AIF1 expression was more than six-fold 24 h after the cut, remained stable around two-fold until day 15 post-amputation, and returned to basal level after day 21 post-treatment (Figure 1C).

As shown in Figure S2, the presence of *Pc*-AIF1 transcripts was confirmed in all the tissues analyzed in this study. Specifically, expression was detected in circulating hemocytes from unchallenged hemolymph, the PK, the control left tentacle at 0 hpa (i.e., non-amputated), the amputated left tentacle during the early regenerative phase (24 hpa), and in both the right (contralateral) and left (ipsilateral) cerebral ganglia. No amplification was observed in the negative control (distilled water), indicating high specificity of the primers for the target sequence. Sequencing of the PCR amplicons confirmed their identity, showing a specific match to the *Pc*-AIF1 sequence deposited in NCBI following alignment (Figure S3).

### 2.2. Pc-AIF1 Is Expressed by Circulating and Resident Hemocytes

**Figure 2:**
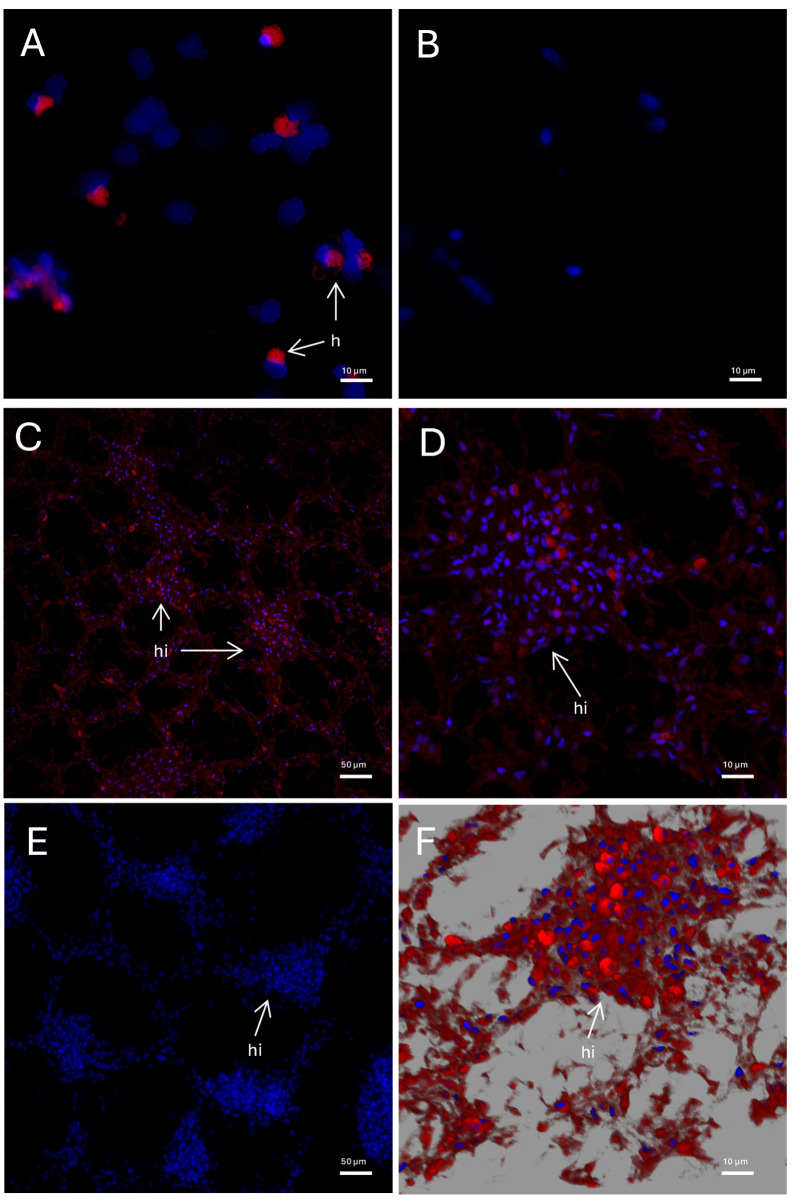
FISH using *Pc*-AIF1 antisense probe on circulating hemocytes and PK. Images have been acquired on a confocal microscope. *Pc*-AIF1 positive hemocytes are depicted in red. Nuclei have been counterstained with DAPI (blue). (**A**) Circulating hemocytes (100× objective); (**B**) Hemocyte negative control, obtained by omitting the probe (40× objective); (**C**) PK at low magnification (20× objective); (**D**) PK at high magnification (40× objective); (**E**) PK negative control (40× objective); (**F**) reconstruction of a PK hemocyte islet obtained from a Z-stack acquisition (40× objective). h = hemocyte; hi = hemocyte islet.

The tissue distribution of *Pc-*AIF1 was further validated through FISH performed on selected target organs. *Pc-*AIF1 signals were detected in the cytoplasm of approximately 12 ± 6% of total circulating hemocytes in unchallenged snails (Figure 2A). In the PK, *Pc-*AIF1 transcripts were predominantly localized within areas corresponding to hemocyte islets, indicating expression in resident hemocytes (Figure 2C,D,F). The stomach, an organ in which hemocytes have never been reported, was used as a negative control and showed no positive signal (Figure S4).

### 2.3. Pc-AIF1 Is Expressed in Cells Surrounding the Control Cerebral Ganglia and by Cells Infiltrating Cephalic Tentacle Blastema

**Figure 3:**
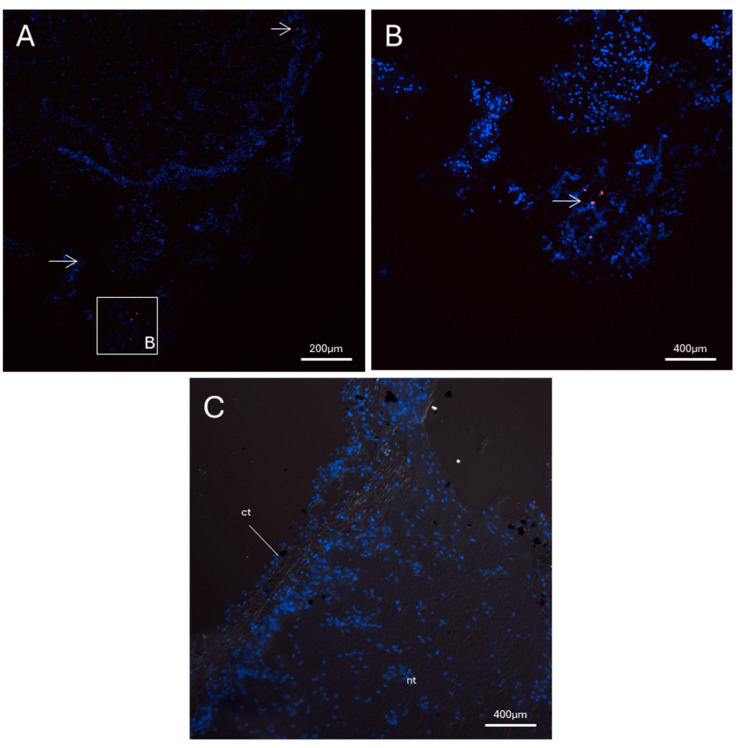
FISH for *Pc*-AIF1 performed on cerebral ganglia from a non-amputated snail. *Pc*-AIF1 positive cells are depicted in red and highlighted with arrows. The nuclei have been counterstained with DAPI (blue). (**A**) Control cerebral ganglia (20× objective). The inset B corresponds to the area represented in panel (**B**); (**B**) Control cerebral ganglia (40× objective); (**C**) negative control of the FISH reaction (no probe). ct = connective tissue; nt = neural tissue.

**Figure 4:**
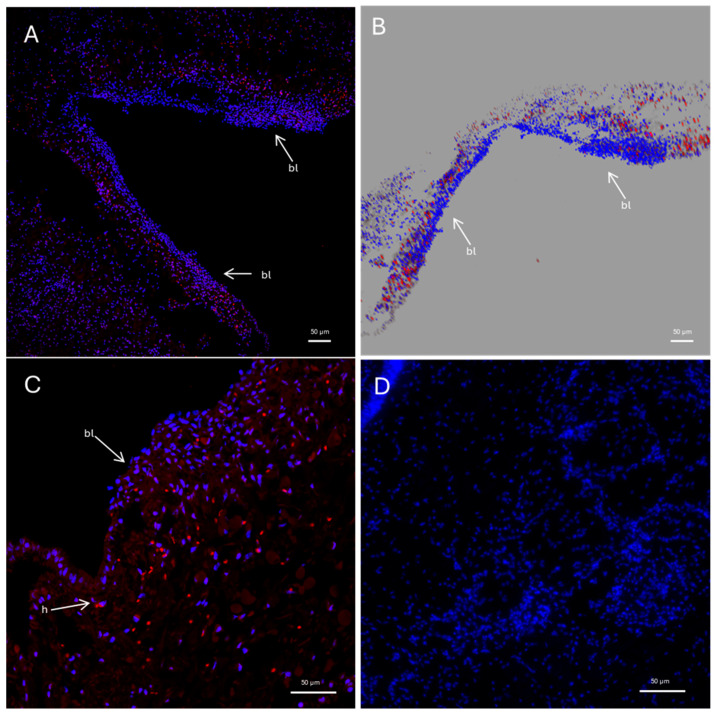
Confocal microscope images of FISH for *Pc*-AIF1 on regenerating cephalic tentacle. Images have been acquired on confocal microscope. *Pc*-AIF1 positive hemocytes are depicted in red. Nuclei have been counterstained with DAPI (blue). (**A**) Newly formed blastema of regenerating tentacle 24 hpa at low magnification (20 × objective); (**B**) reconstruction obtained from a Z-stack acquisition of 24 hpa blastema presented in section (**A**); (**C**) Details of blastema (40× objective); (**D**) uncut left control tentacle (40× objective). bl = blastema; h = hemocytes.

FISH analysis for *Pc*-AIF1 evidenced expression in the cerebral ganglia of *P. canaliculata* (Figure 3). The expression is limited to a few cells localized in the connective tissue surrounding the neural components; no positivity was retrieved in neurons. *Pc*-AIF1 expression was very low or undetectable in the uninjured cephalic tentacles at 0 hpa (Figure 4). In contrast, during the early stages of tentacle regeneration, a visible increase in *Pc*-AIF1-positive cells was observed within the blastema formed at 24 hpa (Figure 4). Laser-scanning confocal microscopy, followed by reconstruction of Z-stack images, showed a distinct spatial distribution of *Pc*-AIF1-expressing cells across the blastema region of the regenerating cephalic tentacle (Figure 4A,B). These positive cells were primarily localized along the lateral margins of the blastema, near the regenerating wound epithelium.

### 2.4. Presence of Microglia-like Cells in P. canaliculata Ganglia

Since AIF-1 is primarily expressed in microglial cells in vertebrates [20,48], and our analyses revealed AIF-1 transcription in several tissues of *P. canaliculata*, positivity to RCA120 was investigated in circulating hemocytes, PK and the blastema of the cephalic tentacle at 24 hpa. RCA120 is a vegetal agglutinin that is used to recognize microglia-like cells in mammals [48] and has previously been used successfully in the mussel *M. galloprovincialis* [49].

**Figure 5:**
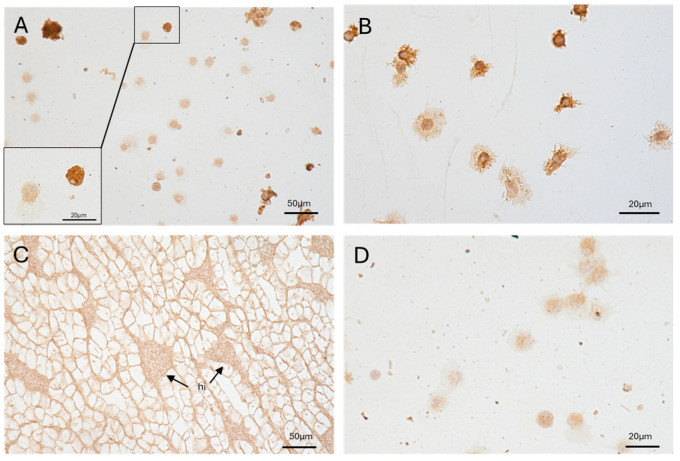
Identification of the RCA120-positive cells among the circulating hemocytes and in the hemocyte islets of the PK. (**A**) Cytocentrifuged hemocytes (Section 4.3) (40× objective) and inset (100× objective); (**B**) spread hemocytes after cell adhesion to a glass slide (100× objective); (**C**) PK containing hemocyte aggregates (40× objective); (**D**) negative control (omitting the RCA120 lectin) of the immunohistochemical reaction (40× objective). hi = hemocyte islets.

Circulating hemocytes showed varying degrees of positivity to RCA120. No RCA120 positivity was found in PK, including in the hemocyte islets (Figure 5).

**Figure 6:**
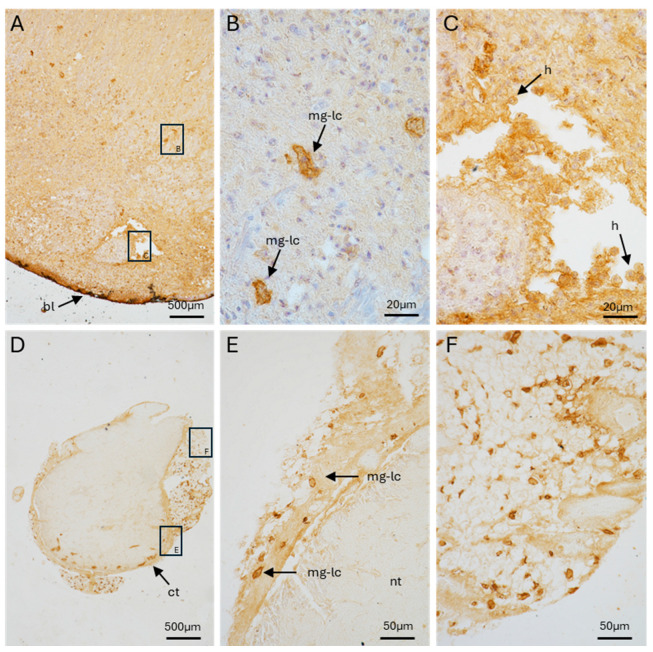
Identification of RCA120-positive cells interspersed within the cephalic tentacle blastema 24 hpa (**A**–**C**) and control ganglia from non-amputated snails (**D**–**F**). (**A**) low magnification of a cephalic tentacle blastema (10×objective). The insets (**B**,**C**) correspond to the areas detailed in panels (**B**,**C**); (**B**) RCA120-positive cells infiltrating the blastema (100×); (**C**) RCA120-positive cells in a blood-containing lacuna (100×); (**D**) low magnification of cerebral ganglia (10×objective). The insets (**E**,**F**) correspond to the areas detailed in panels (**E**,**F**); (**E**,**F**) detailed views of RCA120 positive cells in connective tissue of ganglia (40×) (see Figure S5 for morphological staining). bl = blastema; ct = connective tissue; h = hemocytes; mg-lc = microglia-like cell.

Like the hemocytes, the blastema of regenerating cephalic tentacles exhibited cells with different levels of RCA120 positivity. Some cells are intensely positive to RCA120 and were localized throughout the amputated cephalic tentacle blastema. Other cells in proximity to the blastema, where hemocytes are more frequently found [45], exhibited a weaker positivity (Figure 6A–C). Our microscopy analysis revealed that the inner portion of the ganglia was negative for RCA120 staining. However, RCA120-positive cells similar in shape to those observed in the amputated tentacle were consistently observed in the connective tissue surrounding the neurons of the ganglia (Figure 6D–F).

Regions positive for RCA120 in the cerebral ganglia (Figure S5) were correlated with corresponding transverse sections of the entire head (Figure S6). Additional sections from similar specimens were stained with hematoxylin-eosin (Figures S5A and S7A) or Masson’s trichrome (Figures S5B and S7B) to visualize the localization of microglia-like cells. Similarly, RCA120-positive cells were localized in connective tissue adjacent to nervous structures (i.e., the tentacle, the eye, and the nerve cord) in transverse sections of the entire head (Figures S6 and S7). These results further support the distribution of microglia-like cells within perineural connective tissue, rather than within the ganglia. Negative controls (omitting lectin) are shown in Figure 5D and Figure S8. Moreover, we performed the immunohistochemical reaction on the stomach, i.e., an organ that does not contain hemocytes, and no positive cells were found (Figure S4).

### 2.5. Pc-AIF1 Expression Is Up-Regulated in Cerebral Ganglia During Tentacle Regeneration

**Figure 7:**
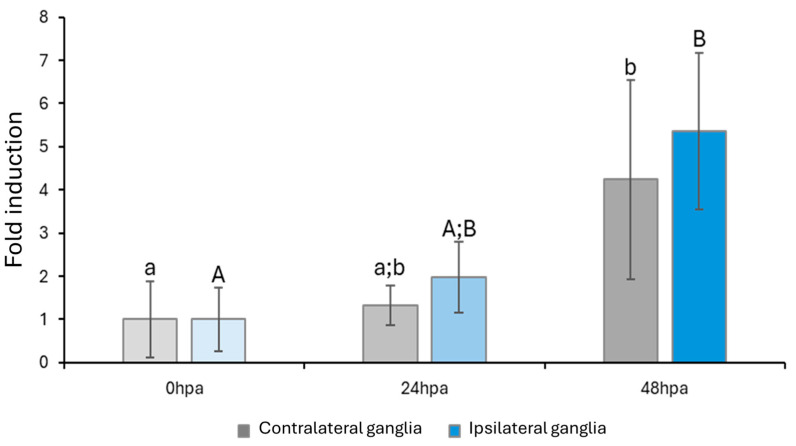
RT-qPCR analysis of *Pc*-AIF1 expression in contralateral (grey) and ipsilateral (blue) cerebral ganglia during the regeneration of the amputated left cephalic tentacle at 24 and 48 hpa. Different lowercase (contralateral) and uppercase (ipsilateral) letters indicate significant differences among the samples in each group (*p* < 0.05).

The expression of *Pc*-AIF1 after left tentacle amputation was assessed in both the left (ipsilateral) and right (contralateral) cerebral ganglia at 24 and 48 hpa using RT-qPCR. (Figure 7). The results revealed a significant upregulation of *Pc*-AIF1 expressions at 48 hpa in both ganglia compared to the control (0 hpa) and the 24 hpa time point. In particular, the expression in the ipsilateral ganglia was over fivefold higher than in uninjured controls (*p* = 0.026), while the contralateral ganglia exhibited a more than fourfold increase (*p* = 0.012). No statistically significant differences were detected between the two ganglia at any of the time points analyzed (*p* > 0.05).

## 3. Discussion

AIF1 is a highly conserved molecule, present both in vertebrates and invertebrates. It plays a key role in inflammatory, immune, and neuro-immune contexts, and it is produced mainly by microglial and macrophagic cells [10,11]. This study examines the expression profile of *Pc*-AIF1 in the tissues of the freshwater gastropod *P. canaliculata*, with a particular focus on its presence in immune-related and neural regenerative tissues.

Analysis of available transcriptomic data reveals that *Pc*-AIF1 is considerably expressed in hemocytes and in tissues with a high concentration of these cells (e.g., the heart). FISH experiments confirmed the expression of *Pc*-AIF1 in the circulating hemocytes of *P. canaliculata*, with approximately 12% of immune cells being positive under physiological conditions. This finding supports the hypothesis that *Pc*-AIF1 is constitutively involved in the immune response of *P. canaliculata*, potentially through a functionally specialized subpopulation of hemocytes dedicated to its expression. In this context, experiments combining image-based flow cytometry with a convolutional neural network have revealed nine different hemocyte clusters in *P. canaliculata* hemolymph. While a link to a specific cell function (e.g., phagocytosis) could be demonstrated for some of these clusters [50], it is reasonable to speculate that the degree of diversity may conceal other subpopulation-specific functions among circulating hemocytes. This pattern is consistent with findings in other mollusks, including bivalves and gastropods, where AIF1 expression is typically localized in immunocytes [26,29]. The presence of AIF1 in hemocytes has been documented in *M. gigas*, *H. discus discus,* and *P. martensii*, where it is constitutively expressed, but strongly inducible in response to immune stimuli [25,26,27]. Notably, a rapid increase in AIF1 expression has been observed in the Pacific oyster following exposure to PAMPs, bacterial infections, or tissue damage, suggesting an active role in the initial stages of acute inflammatory responses [25]. In all these mollusk species, the highest levels of AIF1 are consistently detected in hemocytes, further confirming the molecule’s close association with immune-competent cells in mollusks.

In *P. canaliculata*, *Pc*-AIF1 is also expressed in hemocyte-containing organs, such as the heart and the PK. In PK, *Pc*-AIF1 expression is primarily localized in hemocyte islets (Figure 6), which may serve as aggregates of phagocytic hemocytes or active hematopoietic sites [43]. These aggregates exhibit evident phagocytic activity [51], suggesting that, as observed for other mollusk species, *Pc*-AIF1 may play a role in the responses elicited by pathogens or harmful xenobiotics in *P. canaliculata*.

In lophotrochozoans, the constitutive expression of AIF1 was first identified in microglial cells of the medicinal leech, *H. medicinalis*. In this species, *Hm*-AIF1 is expressed in immune-competent cells that migrate towards inflammatory sites in the initial response to neural injury [21]. Furthermore, as we evidenced in *P. canaliculata*, *Hm*-AIF-1 is constitutively expressed in a macrophage-like subpopulation, and in the leech the recombinant protein acts as a chemoattractant for phagocytic cells and promotes angiogenesis [22]. In invertebrates, the expression levels of AIF1 in hemocytes increase following bacterial infection or tissue damage [26,52,53]. Consequently, AIF1 accumulation in various organs and tissues, as observed during neural repair of *H. medicinalis*, has also been interpreted as resulting from the local infiltration of activated circulating hemocytes [21]. On this basis, the detection of basal *Pc*-AIF1 expression across several non-immune organs may be attributed to the open circulatory system of these organisms, through which hemolymph, and circulating hemocytes, permeate all tissues. Alternatively, this could be due to the presence of tissue-resident immunocytes that are difficult to single out in the absence of reliable markers [45].

In line with observations on the neural repair of *H. medicinalis* [24], available transcriptomic data also indicate that there is a marked increase in *Pc*-AIF1 expression directly within the injured organ during the early stages of the eye regeneration [31]. FISH analysis of the cephalic tentacle blastema confirmed that some cells expressed *Pc*-AIF1 24 hpa. As hemocytes express *Pc*-AIF1 under basal conditions and can be recognized in the tentacle blastema during the initial stages of tentacle regeneration [30], it is reasonable to speculate that the *Pc*-AIF1-positive cells within the blastema are tissue-resident or patrolling hemocytes. The selective and temporary elimination of phagocytic cells using clodronate liposomes alters both the timing of tentacle regeneration and the expression profile of *Pc*-AIF1in the blastema [30], which further supports the hypothesis of an existing link between the distinct functional roles of hemocyte subpopulations reaching the wound and *Pc*-AIF1 expression in neural repair in *P. canaliculata*. In this context, during cephalic tentacle regeneration, a peak in *Pc*-AIF1 expression in ipsilateral and contralateral ganglia was observed at 48 hpa, in line with the ganglionic transcriptional response registered during ocular regeneration. The nerve cord connecting the two cerebral ganglia could represent the anatomical component that supports the bilateral response to monolateral amputation. A similar scenario has been described during neural repair of *H. medicinalis*, where *Hm*-AIF1 is expressed in the central nervous system under basal conditions and is dramatically up-regulated within 48 h of injury or tissue transplantation [22,24]. Immunohistochemical analyses have confirmed the expression of *Hm*-AIF1 in activated microglial cells, which accumulate significantly at injury sites, along connective fibers, and around neuronal cell bodies within a few days [22,23]. To determine whether *Pc*-AIF1 expression registered by RT-qPCR in *P. canaliculata* neural components could also be attributed to microglia-like cells, immunohistochemical analyses were conducted on selected target organs using RCA120, a vegetal lectin already used in vertebrates and in the bivalve *M. galloprovincialis* to recognize microglial and potential microglial-like cells, respectively [48,49]. Evident RCA120 labeling was observed in a small subset of circulating hemocytes, while no RCA120-positive cells were detected among the resident hemocytes in renal hemocyte aggregates. This observation (Figure 5) supports the findings that various subsets of circulating hemocytes exist [50] and that the hemocytic aggregates in the PK contain a distinct population of phagocytic hemocytes [43]. Consistent with the finding that RCA120 marks specific hemocytes, RCA120-positive cells were identified in the cephalic tentacle and cerebral ganglia, within the connective tissue surrounding neural structures. A specific pattern of positivity to the lectin was observed in the regenerating blastema (Figure 6): a weak signal was detected in cells that may correspond to tissue-resident hemocytic cells, while a stronger signal was present in cells that may represent microglia-like cells distributed around the cerebral ganglia and within the nerve-associated connective tissue. In the cerebral ganglia, RCA120-positive cells were found in the peripheral connective tissue, in a similar location to the *Pc*-AIF1-positive cells identified using FISH. In addition to being negative to *Pc-*AIF1 by FISH, ganglionic neurons were also negative for RCA120 by immunocytochemical staining. These findings suggest that the *Pc*-AIF1 expression, as determined by transcriptomic analysis and qPCR experiments, is primarily due to circulating and tissue-resident immunocytes and that RCA120-positive cells correspond to microglia-like cells. However, co-localization experiments remain necessary to confirm that RCA120-positive microglia-like cells also express *Pc*-AIF1. Nevertheless, as it has been suggested in leech, the *Pc-*AIF1-expressing cells could represent a functional equivalent of vertebrate microglia, which is involved in inflammatory responses and neural regeneration [54,55,56,57]. Notably, AIF1 has been implicated in inducing pro-inflammatory cytokine expression, which may play a critical role in regulating regenerative processes [13,14,58]. The expression of *Pc*-AIF1 in immune-competent cells and in cells associated with the nervous system of *P. canaliculata* is consistent with the well-established cooperation between the immune and nervous systems that is essential for maintaining neuronal homeostasis and promoting neural repair [59,60,61]. The immune and neuroendocrine systems are known to share a wide range of signaling molecules, including cytokines, complement factors, receptors, and neurotransmitters, and this molecular overlap reflects their profound functional interconnection despite their distinct anatomical organization [62,63]. A growing body of evidence suggests that immune and neuronal cells interact closely with each other in invertebrates, too [64]. In adult crayfish, for instance, immune cells promote neurogenesis in both normal conditions and in response to brain injury. Following neurodegeneration, hemocytes are actively recruited to neurogenic niches, where they influence nitric oxide signaling and induce vascular remodeling [65]. In *Procambarus clarkii*, adult olfactory neurons regenerate through the transdifferentiation of circulating hemocytes that progressively acquire neuronal characteristics [66]. This phenomenon has also sparked renewed interest in neuronal transdifferentiations in humans [67]. Another example comes from *Drosophila melanogaster*, where the behavior of larval hemocytes is regulated by subpopulations of neurons. Signals from these neurons guide hemocyte migration back to hematopoietic pockets, demonstrating direct neuro-immune control of hemocyte mobilization and homing [68]. The details concerning the biological roles of *Pc*-AIF-1 remain unknown. However, its main expression in circulating hemocyte and hemocyte-rich organs, its expression in cells that, for their localization, may represent microglia-like cells, and its increased expression in cerebral ganglia components during tentacle regeneration, which recalls the transcriptomic data available for eye regeneration [31], allow us to speculate that *Pc*-AIF-1 may be characteristic of the hemocyte pro-inflammatory subpopulation in the immediate response to injury and may contribute to microglia-like cell activation during the early regeneration of the cephalic tentacle. While the synthesis of *Pc*-AIF1 by ganglionic neurons cannot be ruled out in our experimental setting, the data obtained by FISH suggest that, as for vertebrates, ganglionic neurons do not directly produce *Pc*-AIF1 [2,3,4]. This indicates that the involvement of the cerebral ganglia in cephalic tentacle regeneration may be mediated by other soluble factors. It also remains to be determined whether the time profile of the increase in *Pc*-AIF1 expression registered in the ganglia depends on specific signalling from accumulated hemocytes diffusing in the extracellular fluid and reaching the microglia-like cells surrounding the cerebral ganglia, or if other cellular processes are involved, such as retrograde signalling after neural injury, which activates new transcription and synthesis of signal molecules that may impact regeneration and microglia activation [63,69,70,71].

## 4. Materials and Methods

### 4.1. Samples Retrieval and Whole Transcriptome Expression Analysis

A total of 316 whole transcriptome (RNA-seq) samples of *P. canaliculata* were retrieved from the NCBI Short Read Archive (SRA, accessed the 1 March 2025) using *srahunter* [72], encompassing 33 different tissue categories as detailed in the associated metadata (Table S1). A read-quality trimming step was performed to remove low-quality bases and adaptors using *fastp* v0.23.1 [73] with the following parameters: -V -w 16 -x -g -n 2 -5 -3 -p -l 75. Trimmed reads were mapped on the *P. canaliculata* reference genome (NCBI ID: GCF_003073045.1) with the CLC mapper (CLC Genomic Workbench, Qiagen, US) applying the following parameters: Mismatch cost = 2; Insertion cost = 3; Deletion cost = 3; Length fraction = 0.8; Similarity fraction = 0.8. A single sample (SRR1616966) was discharged from the following analyses because of the low mapping rate (<10%), resulting in a final dataset composed of 315 RNA-seq samples. The expression values of the 24,194 genes annotated in the reference genome were computed as Transcripts Per Million (TPM) to normalize over samples characterized by different sequencing depths (Figure S1). The expression values related to the *P. canaliculata* AIF-1 gene (Gene ID: LOC112566076, protein ID: XP_025097806.1) were extracted and plotted by tissue and/or condition using the tidyverse, ggplot2, plyr, and ggpubr packages implemented in R v4.2.1.

### 4.2. Animals

*P. canaliculata* specimens are bred in the authorized aquatic facility of the Department of Life Sciences of the University of Modena and Reggio Emilia since 2008. For these experiments the snails were bred in dechlorinated tap water at a controlled temperature of 25 ± 1 °C under a 12-h light/dark cycle. Twice a week, approximately two-thirds of the tank water was exchanged. Following each water change, the snails were fed with a mix of leafy greens commonly consumed by humans. Only adult snails (age > 1.5 years) were selected for the experiments described below. To minimize the presence of residual food or digestive by-products, the animals underwent a 48-h fasting period prior to hemolymph extraction or organ dissection.

### 4.3. Treatment, Animal Dissection, and Organ Collection

Hemolymph was collected from control individuals by gently applying continuous pressure to the operculum. The released hemolymph was gathered into 10 mL tubes kept on ice, with approximately 2 mL obtained from each specimen. For each slide preparation, 250 μL of freshly collected hemolymph was either loaded into a Cytospin II™ (Shandon Inc., Pittsburgh, PA, USA) and centrifuged at 400 rpm for 3 min or deposited onto slides to permit cell adhesion for 10 min. The hemocytes were then immediately fixed with 4% paraformaldehyde (pFA) in 1× PBS for 3 min at room temperature. Fixation was halted by rinsing the slides in 1× PBS for 5 min before either being processed for FISH or undergoing an immunohistochemistry procedure.

For organ dissection, control snails were anesthetized in granular ice for 30 min then PKs, stomach, and both the cerebral ganglia were collected, sacrificing the animals.

The left cephalic tentacles were amputated from the animals after they had been anesthetized in granular ice for 30 min. The cephalic tentacles were cut at the base [45]. After amputation, the snails were kept outside the water and at room temperature for 30 min before being placed individually in standard maintenance tanks to recover. The cerebral ganglia and the blastema of cephalic tentacle were collected during regeneration after 0, 24, 48 hpa (after anesthetization). The collected organs were fixed in 4% pFA overnight at 4 °C or in freshly prepared Bouin’s solution. The fixed tissue samples were washed in 70% ethanol and kept in clean 70% ethanol for storage before the clarification, inclusion, and sectioning steps.

Furthermore, pelleted hemocytes (400× *g* for 5 min), PK, control and regenerating cephalic tentacles (0 and 24 hpa), left and right cerebral ganglia from non-amputated snails, left and right cerebral ganglia from left tentacle-amputated snails (0, 24, 48 hpa) were weighted and stored at −80 °C for subsequent RNA extraction and gene expression analysis (maximum 5 mg of each organ for one extraction).

### 4.4. RT-PCR, Gel Extraction, and Sequencing

Total RNA purification was performed with ReliaPrep™ RNA Miniprep Systems (Promega, Madison, WI, USA) following the manufacturer’s protocol. All RNA samples were then checked for purity and quantified with a NanoDrop™ (ND1000 Spectrophotometer, Thermo Fisher Scientific, Waltham, MA, USA). Approximately 1 µg of RNA was reverse transcribed to cDNA using the iScript cDNA Synthesis Kit (Bio-Rad Laboratories, Inc., Hercules, CA, USA) according to the manufacturer’s instructions.

The obtained cDNAs, corresponding to hemocytes, PK, cephalic tentacles (0 and 24 hpa), right and left control ganglia, have been employed as templates (1 μL) in RT-PCR reactions using GoTaq^®^ DNA Polymerase (Promega, Madison, WI, USA). Reactions with a final volume of 25 µL were prepared containing: 1× GoTaq^®^ Reaction Buffer, 0.2 mM each dNTPs, 0.5 µM of forward and reverse *Pc*-AIF1 primers (Table S2), 1.25 U of GoTaq^®^ polymerase (0.125 µL each tube). The thermal cycling conditions applied were: initial denaturation at 95 °C for 2 min; followed by 35 cycles of 95 °C for 30 s, 58° for 30 s, and 72 °C for 1 min; with a final extension at 72 °C for 5 min. RT-PCR employing *Pc-AIF1* primers for riboprobe synthesis purpose required an annealing temperature of 55 °C (Table S2). Amplicons were separated by electrophoresis on a 1.5% agarose gel stained with ethidium bromide. Bands of the expected size were excised and purified using GenElute Gel Extraction Kit (Sigma-Aldrich, Burbank, CA, USA) according to the manufacturer’s protocol. Purified PCR products were quantified using a Nanodrops™ and sequenced using Sanger sequencing (Mix2seq, Eurofins Genomics, Ebersburg, Germany).

### 4.5. Slide Preparation and Staining for Histological Analysis

Fixed samples stored in 70% ethanol underwent dehydration through an ascending scale of graded ethanol series (70%, 95%, 100%), cleared with xylene, and were embedded in Paraplast^®^. The Paraplast^®^-embedded tissues were sectioned into 7 µm slices using a rotary microtome and mounted onto StarFrost^®^ glass slides. Prior to histological technique, standard rehydration procedures through a series of ethanol solutions (100%, 95%, 70%), followed by clearing with 100% xylene, as described in Ferri et al., 2024 [74]. Slides were processed for FISH or Immunohistochemistry or stained with Mayer’s Hematoxylin and Eosin (HE) and Masson’s trichrome staining (Bio-Optica, Milan, Italy).

### 4.6. Riboprobe Synthesis

Purified *Pc*-AIF1 amplicons (580 bp) from hemocytes’ cDNA were ligated into pGEM^®^-T Easy Vector Systems plasmid (Promega, Madison, WI, USA) following the protocol described in the technical manual. A molecular ratio insert:vector adopted was 3:1 (*Pc*-AIF1 insert (28 ng), 1 µL of pGEM^®^-T Easy Vector Systems (50 ng)). Ligation was performed in 0.5 mL tubes incubating reactions 1 h at room temperature. Two µL of the ligation reaction were used to transfect 50 µL of JM109 High Efficiency Competent Cells (Promega, Madison, WI, USA) by thermal shock. Transformed cells were added to 950 µL of SOC medium, incubated for 90 min at 37 °C, and then 100 µL spread on prewarmed LB/ampicillin/IPTG/X-Gal plates ((LB: NaCl 6 M, bacto-tryptone 1%, yeast extract 0.5%, agar 2%, pH 7); ampicillin (100 μg/mL), X-gal (20 mg/mL)). Plates were incubated overnight at 37 °C to allow the selection of transformed colonies. White colonies were selected and tested for insert presence by colony-PCR using specific *Pc*-AIF1, T7 and Sp6 primers (Table S2). The reactions were performed according to the datasheet for GoTaq^®^ DNA Polymerase, using an annealing temperature of 50 °C. The correct size and orientation of inserts was verified by separating the PCRs product in 1.5% agarose gel. Positive corresponding colonies were incubated overnight at 37 °C in bacterial culture tubes containing 2.5 mL of LB broth supplemented with ampicillin (100 μg/mL). Transformed colonies have been then processed for plasmid extraction using the commercial kit PureYield™ Plasmid Miniprep System (Promega, Madison, WI; USA) according to the manufacturer’s instructions. Purified plasmids containing correctly oriented inserts have been used as templates (20 ng) in PCR using T7 or Sp6 primer as already described. Resulting bands were purified then used (1 μg) as template in transcription reactions assembled as follows: 12.5 μL PCR product; 5 μL di Trans Buffer 5X; 2.5 μL of 10X rNTPs 2.5 mM; 2.5 μL of DIG-UTP (200 ng/μL); 1 μL RNAse inhibitor (80 U/μL); 1.5 μL of either T7 or Sp6 polymerase. Reactions were performed for 4 h at 37 °C. Sense and antisense *Pc*-AIF1 RNA probes were DNAse I digested (1 h at 37 °C), then purified by treatment with 3M sodium acetate followed by precipitation in ice-cold 100% ethanol and incubation at −80 °C for 30 min. Probes were centrifuged at maximum speed 30 min at 4 °C, washed with ethanol 70% and then resuspended in 100 μL of deionized formamide. The concentration of the probes was determined by semi-quantitative analysis of the probe bands after electrophoresis in an agarose gel using Bio-Rad Quantity One^®^ software (version 4.5.2), and was adjusted to 10 ng/μL.

### 4.7. FISH Protocol

FISH was performed on samples obtained from three snails. For each sample, we prepared at least 10 slides, each containing 10 tissue sections. FISH was carried out on cytocentrifuged hemocyte slides prepared as described in Section 4.3 and on rehydrated tissue sections (including PK, cephalic tentacle, and cerebral ganglia) as described in Section 4.5, using a modified version of the protocol originally described by King and Newmark [75]. Slides were first bleached in a solution containing 1.2% hydrogen peroxide and 5% formamide in 0.5× SSC for one hour to enhance probe accessibility. Following this, the samples were rinsed twice with 1× PBS containing 0.1% Tween^®^ 20 (PBST; Bio-Rad Laboratories, Inc.), treated with 3 µg/mL proteinase K in PBST for 10 min to permeabilize the tissue, and then post-fixed in 4% pFA for another 10 min. Subsequently, the slides were incubated in a 1:1 mixture of 1× PBS and Pre-Hybridization Solution (HS) for 10 min at room temperature. This was followed by incubation in HS alone (containing 50% formamide, 1% Tween^®^ 20, 5× SSC, 1× Denhardt’s solution, 50 mM dithiothreitol, 1 mg/mL torula yeast RNA, and 100 µg/mL heparin) for 1 h at 58 °C. Hybridization was performed overnight at 58 °C using *Pc*-AIF1 DIG-labeled riboprobes (50 ng/µL), diluted 1:500 in HS. Control slides were processed identically but without the addition of probes. The specificity of the *Pc*-AIF1 DIG-labeled riboprobes was also assessed by testing them on the stomach, an organ that is not characterized by the presence of hemocytes (Figure S4). Post-hybridization washes included two 10 min rinses in Wash-HS (50% formamide, 1% Tween^®^ 20, 5× SSC, 1× Denhardt’s) at 58 °C, followed by two 10 min washes in a 1:1 mix of Wash-HS and 2× SSC at the same temperature. This was followed by three 10 min washes in 2× SSC with 0.1% Tween^®^ 20, and three additional 10 min washes in 0.2× SSC with 0.1% Tween^®^ 20, all at 58 °C. Slides were then washed twice with maleic acid buffer containing Tween^®^ 20 (MABT: 11.61 g/L maleic acid, 150 mM NaCl, 0.1% Tween^®^ 20) at room temperature. To block nonspecific binding, the samples were incubated for 1 h at room temperature in a blocking solution composed of 5% horse serum and 0.5% Roche Western Blocking Reagent in MABT. This was followed by overnight incubation at 4 °C with an anti-DIG-peroxidase-conjugated antibody (1:1000; Roche) diluted in filtered blocking solution. The following day, the slides were washed six times for 20 min each in MABT at room temperature. Signal detection was performed using a tyramide signal amplification system. Slides were incubated for 1 h at room temperature with the Tyramide Development Solution (TDS), consisting of 1:200 fluorescein amidite (FAM)-tyramide in borate buffer (2 M NaCl, 0.1 M boric acid) supplemented with 0.06‰ hydrogen peroxide. Excess TDS was removed with two PBST washes at room temperature. Finally, nuclear staining was carried out with DAPI (1 μg/mL), which was added directly to the Mowiol^®^ mounting medium used.

### 4.8. Immunohystochemistry

The immunohistochemical protocol followed was based on a method previously described by Ottaviani and Cossarizza [76]. After endogenous peroxidase blocking, hemocytes and tissue sections were incubated overnight at 4 °C with biotinylated *Ricinus communis* agglutinin I (RCA 120, Vector) at a dilution of 1:1000, a marker for microglial cells in vertebrates [49]. Detection of immunoreactivity was carried out using the avidin–biotin–peroxidase complex method (Vectastain ABC-HRP Kit, Vector Laboratories, Burlingame, CA, USA), with diaminobenzidine (DAB) serving as the chromogenic substrate. Appropriate negative controls, on the stomach section (Figure S4) or omitting lectin (Figure S8), were included to ensure specificity.

### 4.9. Imaging Acquisition

Images from FISH were taken with a Leica TCS SP8 confocal microscope (Leica Microsystems Srl, Milan, Italy) at the Centro Interdipartimentale Grandi Strumenti (C.I.G.S.) of the University of Modena and Reggio Emilia, Modena, Italy. Images from histology and immunohistochemistry were taken with a Nikon Eclipse Ni-E microscope (Leica Microsystems Srl, Milan, Italy) at the National Biodiversity Future Centre (NBFC) of the University of Modena and Reggio Emilia, Modena, Italy. All other image processing was performed with the ImageJ software (version number 1.52p, NIH, Bethesda, MD, USA).

### 4.10. RT-qPCR and Expression Analysis

Quantitative PCR (qPCR) was carried out using the SsoAdvanced™ Universal SYBR^®^ Green Supermix (Bio-Rad Laboratories, Inc.), along with specific primers targeting the reference gene ribosomal protein L5 (*Pc*-RpL5) and *Pc*-AIF1 (see Table S2). The reactions followed the manufacturer’s recommended protocol. The thermal cycling conditions included an initial denaturation at 95 °C for 2 min (1 cycle), followed by 30 cycles of denaturation at 95 °C for 10 s and annealing/extension at 60 °C for 30 s. Each reaction was performed in triplicate using the CFX-Duet Real-Time PCR Detection System (Bio-Rad Laboratories, Inc.) at the NBFC of the University of Modena and Reggio Emilia, Modena, Italy. Upon completion, melting curve analysis was conducted to verify the specificity of the amplified products.

Gene expression was analyzed using the 2^−ΔΔCt^ method [77]. Results are presented as fold changes, with the control group set as the reference value (fold change = 1). Expression levels were normalized to the reference gene *Pc*-RpL5 and presented as relative expression ratios. To assess the normality and homogeneity of variances, the Shapiro–Wilk test and the F-test for homoscedasticity were performed (*p* > 0.05). Subsequently, qPCR data were analyzed by one-way ANOVA followed by Tukey’s post-hoc test using PAST statistical software [78]. Differences were considered statistically significant at *p* < 0.05.

## 5. Conclusions

This study characterized the expression and cellular distribution of *Pc*-AIF1 in *P. canaliculata*. The results revealed that *Pc*-AIF1 is constitutively expressed, and it is involved in the immune response to wounding and during regenerative processes. The analysis of deposited transcriptomic datasets and locally produced experimental data indicates that *Pc*-AIF1 is maximally expressed in circulating hemocyte subpopulations and in the cerebral ganglia, where immunocytochemical experiments indicated the presence of potential microglia-like cells. During cephalic tentacle regeneration, *Pc-*AIF1 expression increases in a temporally regulated manner in the cerebral ganglia, suggesting an association between *Pc*-AIF1 and neuroimmunity. Nevertheless, the limited availability, or the absence, of RNA-seq samples for certain tissues is a limitation that should be addressed in future studies to improve the expression information for this novel model species. Further studies are also needed to determine whether *Pc*-AIF1 could be a key marker for timing the interaction between the immune and nervous systems during the response to injury and the regeneration of sensory organs in *P. canaliculata*.

## Data Availability

The original contributions presented in this study are included in the article/Supplementary Material. Some of the data presented in this study are available in NCBI Stort Read Archive (SRA, accessed the 1 March 2025).

## Supplementary Materials

The following supporting information can be downloaded at: https://www.mdpi.com/article/10.3390/ijms26189022/s1.
