# Supplementary material for: Pc-AIF1 Is Expressed in Hemocyte-Rich and Neural Tissues and Links Immune Response and Regeneration in the Snail Model Pomacea canaliculata
Source: Int J Mol Sci. 2025 Sep 16;26(18):9022. doi: 10.3390/ijms26189022 (PMC12469834; doi:10.3390/ijms26189022)
Supplement: Supplementary file 1 [file ijms-26-09022-s001.zip › ijms-3759308-supplementary.pdf]

Table S1 Metadata associated with the considered RNA sequencing experiments. For each sample (Run), details about the read length, number of sequenced bases, Bioproject ID, research center are reported together with data related to the sequence sample (library and sample name, tissue, condition and developmental stage). The table has been retrieved from the NCBI SRA database.
Abbreviation for CenterName:1 Hong Kong Baptist University; 2 Stowers Institute For Medical Research; 3 Pearl River Fisheries Research Institute .Cafs; 4 Hunan Agricultural University; 5 Nanjing Forestry University; 6 Agricultural Genomics Institute At Shenzhen, Chinese Academy Of Agricultural Sciences; 7 Yancheng Teacher University; 8 South China Agricultural University; 9 Sanchez Alvarado Lab, Stowers Institute For Medical Research; 10 Guangxi Normal University; 11 Institute Of Zoology, Chinese Academy Of Sciences; 12 Sun Yat-Sen University; 13 National Institute Of Parasitic Diseases, Chinese Center For Disease Control And Prevention; 14 China National Rice Research Institute; 15 Department Of Biology, Hong Kong Baptist Universit.

Run	AvgSpotLen	Base	BioProject	BioSample	CenterName	LibraryName	SampleName	Tissue	Condition	DevStage
SRR6395538	250	6299944250	PRJNA423172	SAMN08213143	1	Pca_AG	Pca_AG	albumen gland		Adult
SRR7224675	101	2765366062	PRJNA473253	SAMN09269458	2	Ampulla	Ampulla	Ampulla		Adult
SRR1616966	200	6568178200	PRJNA264138	SAMN03114858	3	run1	Pomacea canaliculata 1	body		
SRR1616967	200	1717514600	PRJNA264139	SAMN03114859	3	run1	Pomacea canaliculata 2	body		
SRR1616968	200	1228311400	PRJNA264139	SAMN03114864	3	run2	Pomacea canaliculata 3	body		
SRR1616969	200	1414971000	PRJNA264139	SAMN03114865	3	run3	Pomacea canaliculata 4	body		
SRR7216576	300	6596142900	PRJNA473031	SAMN09258470	1	Pca_CT	Pca_CT	cephalic tentacle		Adult
SRR6395539	250	5632926750	PRJNA423172	SAMN08213144	1	Pca_DG	Pca_DG	digestive gland		Adult
SRR26618509	300	6011141700	PRJNA1033406	SAMN38032538	4	P_hep_1	Pomacea canaliculate-1	digestive gland		adult
SRR17245043	300	12590304300	PRJNA788380	SAMN23968665	5	Con4	Con4	digestive gland		
SRR17245044	300	12236827500	PRJNA788380	SAMN23968664	5	Con3	Con3	digestive gland		
SRR17245045	300	12444673200	PRJNA788380	SAMN23968663	5	Con2	Con2	digestive gland		
SRR17245046	300	13307845800	PRJNA788380	SAMN23968662	5	Con1	Con1	digestive gland		
SRR17245047	300	12416591400	PRJNA788380	SAMN23968661	5	Ca6	Ac6	digestive gland		
SRR17245048	300	12518478300	PRJNA788380	SAMN23968660	5	Ca5	Ac5	digestive gland		
SRR17245049	300	12350838300	PRJNA788380	SAMN23968659	5	Ca4	Ac4	digestive gland		
SRR17245050	300	12394921500	PRJNA788380	SAMN23968658	5	Ca3	Ac3	digestive gland		
SRR17245051	300	12952493100	PRJNA788380	SAMN23968667	5	Con6	Con6	digestive gland		
SRR17245052	300	12567816600	PRJNA788380	SAMN23968666	5	Con5	Con5	digestive gland		
SRR17245053	300	12719871900	PRJNA788380	SAMN23968657	5	Ca2	Ac2	digestive gland		
SRR17245054	300	13094531100	PRJNA788380	SAMN23968656	5	Ca1	Ac1	digestive gland		
SRR6429145	286	7475826606	PRJNA427478	SAMN08242144	6	Dg1	Dg1	digestive gland	biologilca replicate 1	
SRR6429146	284	7416078195	PRJNA427478	SAMN08242145	6	Dg2	Dg2	digestive gland	biologilca replicate 2	
SRR6429153	284	2316059965	PRJNA427478	SAMN08242146	6	Dg3	Dg3	digestive gland	biologilca replicate 3	
SRR30483092	300	8479110000	PRJNA1153304	SAMN43388747	7	P.canaliculataDRY151	DRY15-1	digestive gland		
SRR30483093	300	8501737500	PRJNA1153304	SAMN43388746	7	P.canaliculataDRY053	DRY05-3	digestive gland		
SRR30483094	300	8625246300	PRJNA1153304	SAMN43388745	7	P.canaliculataDRY052	DRY05-2	digestive gland		
SRR30483095	300	9219311400	PRJNA1153304	SAMN43388744	7	P.canaliculataDRY051	DRY05-1	digestive gland		
SRR30483096	300	9139848900	PRJNA1153304	SAMN43388743	7	P.canaliculataDRY013	DRY01-3	digestive gland		
SRR30483097	300	9645177600	PRJNA1153304	SAMN43388742	7	P.canaliculataDRY012	DRY01-2	digestive gland		
SRR30483098	300	9071770500	PRJNA1153304	SAMN43388741	7	P.canaliculataDRY011	DRY01-1	digestive gland		
SRR30483099	300	8431938000	PRJNA1153304	SAMN43388740	7	P.canaliculataCK3	CK-3	digestive gland		
SRR30483100	300	8859277500	PRJNA1153304	SAMN43388755	7	P.canaliculataRCY013	RCY01-3	digestive gland		
SRR30483101	300	10160083800	PRJNA1153304	SAMN43388754	7	P.canaliculataRCY012	RCY01-2	digestive gland		
SRR30483102	300	10995954900	PRJNA1153304	SAMN43388753	7	P.canaliculataRCY011	RCY01-1	digestive gland		
SRR30483103	300	8505747000	PRJNA1153304	SAMN43388752	7	P.canaliculataDRY303	DRY30-3	digestive gland		
SRR30483104	300	12145117500	PRJNA1153304	SAMN43388751	7	P.canaliculataDRY302	DRY30-2	digestive gland		
SRR30483105	300	6148244700	PRJNA1153304	SAMN43388750	7	P.canaliculataDRY301	DRY30-1	digestive gland		
SRR30483106	300	7119866400	PRJNA1153304	SAMN43388749	7	P.canaliculataDRY153	DRY15-3	digestive gland		
SRR30483107	300	9555372600	PRJNA1153304	SAMN43388748	7	P.canaliculataDRY152	DRY15-2	digestive gland		
SRR30483108	300	8730753600	PRJNA1153304	SAMN43388739	7	P.canaliculataCK2	CK-2	digestive gland		
SRR30483109	300	8983773000	PRJNA1153304	SAMN43388738	7	P.canaliculataCK1	CK-1	digestive gland		
SRR31174510	302	8754183324	PRJNA1178854	SAMN44484600	8	S_20	CFL4	digestive gland		
SRR31174511	302	7139296610	PRJNA1178854	SAMN44484599	8	S_19	CFL3	digestive gland		
SRR31174512	302	6577756602	PRJNA1178854	SAMN44484598	8	S_18	CFL2	digestive gland		
SRR31174513	302	6639929342	PRJNA1178854	SAMN44484597	8	S_17	CFL1	digestive gland		
SRR31174516	302	8611784284	PRJNA1178854	SAMN44484596	8	S_4	SFL4	digestive gland		
SRR31174523	302	8550370168	PRJNA1178854	SAMN44484616	8	S_24	CML4	digestive gland		
SRR31174524	302	7875217760	PRJNA1178854	SAMN44484615	8	S_23	CML3	digestive gland		
SRR31174525	302	9309730142	PRJNA1178854	SAMN44484614	8	S_22	CML2	digestive gland		
SRR31174526	302	6502356262	PRJNA1178854	SAMN44484613	8	S_21	CML1	digestive gland		
SRR31174527	302	7665883440	PRJNA1178854	SAMN44484595	8	S_3	SFL3	digestive gland		
SRR31174528	302	6755715236	PRJNA1178854	SAMN44484612	8	S_8	SML4	digestive gland		
SRR31174529	302	6843977756	PRJNA1178854	SAMN44484611	8	S_7	SML3	digestive gland		
SRR31174530	302	7223140568	PRJNA1178854	SAMN44484610	8	S_6	SML2	digestive gland		
SRR31174531	302	7171251230	PRJNA1178854	SAMN44484609	8	S_5	SML1	digestive gland		
SRR31174538	302	7737886582	PRJNA1178854	SAMN44484594	8	S_2	SFL2	digestive gland		
SRR31174539	302	6818690994	PRJNA1178854	SAMN44484593	8	S_1	SFL1	digestive gland		
SRR6793906	300	24744626100	PRJNA432822	SAMN08462630	8	Transcriptomic analysis gene expression in the developing embryo	Transcriptome analysis the embryo development of Pomacea canaliculata	embryo		
SRR5229980	300	24744626100	PRJNA362500	SAMN06240709	8	Pomacea canaliculata	Transcriptome data in the developing embryo of Pomacea canaliculata	embryo		
SRR7216573	200	9470925000	PRJNA473031	SAMN09258474	1	Pca_E12	Pca_E12	embryo		
SRR7216574	200	7171428200	PRJNA473031	SAMN09258472	1	Pca_E6	Pca_E6	embryo		days 6
SRR7216575	200	7476798400	PRJNA473031	SAMN09258473	1	Pca_E9	Pca_E9	embryo		days 9
SRR7216577	200	7816399000	PRJNA473031	SAMN09258471	1	Pca_E5	Pca_E5	embryo		days 5
SRR6429147	282	7321199347	PRJNA427478	SAMN08242142	6	De2	De2	embryo	biologilca replicate 2	
SRR6429148	285	7436571598	PRJNA427478	SAMN08242143	6	De3	De3	embryo	biologilca replicate 3	
SRR6429150	287	7477189358	PRJNA427478	SAMN08242141	6	De1	De1	embryo	biologilca replicate 1	
SRR25525693	102	2011839534	PRJNA1002268	SAMN36843224	9	GSM7681314	GSM7681314	eye stalk		28
SRR25525694	102	1983388368	PRJNA1002268	SAMN36843224	9	GSM7681314	GSM7681314	eye stalk		28
SRR25525695	102	1898900340	PRJNA1002268	SAMN36843225	9	GSM7681313	GSM7681313	eye stalk		28
SRR25525696	102	1896849732	PRJNA1002268	SAMN36843225	9	GSM7681313	GSM7681313	eye stalk		28
SRR25525697	102	1725665376	PRJNA1002268	SAMN36843226	9	GSM7681312	GSM7681312	eye stalk		28
SRR25525698	102	1756791594	PRJNA1002268	SAMN36843226	9	GSM7681312	GSM7681312	eye stalk		28
SRR25525699	102	1745722962	PRJNA1002268	SAMN36843227	9	GSM7681311	GSM7681311	eye stalk		28

SRR25525700	102	1843776276	PRJNA1002268	SAMN36843227	9	GSM7681311	GSM7681311	eye stalk		28
SRR25525701	102	1727023914	PRJNA1002268	SAMN36843228	9	GSM7681310	GSM7681310	eye stalk		21
SRR25525702	102	1730098908	PRJNA1002268	SAMN36843228	9	GSM7681310	GSM7681310	eye stalk		21
SRR25525703	102	2014446042	PRJNA1002268	SAMN36843229	9	GSM7681309	GSM7681309	eye stalk		21
SRR25525704	102	2004838152	PRJNA1002268	SAMN36843229	9	GSM7681309	GSM7681309	eye stalk		21
SRR25525705	102	1831347576	PRJNA1002268	SAMN36843230	9	GSM7681308	GSM7681308	eye stalk		21
SRR25525706	102	1871552202	PRJNA1002268	SAMN36843230	9	GSM7681308	GSM7681308	eye stalk		21
SRR25525707	102	1871893902	PRJNA1002268	SAMN36843231	9	GSM7681307	GSM7681307	eye stalk		21
SRR25525708	102	1857891852	PRJNA1002268	SAMN36843231	9	GSM7681307	GSM7681307	eye stalk		21
SRR25525709	102	1872987138	PRJNA1002268	SAMN36843232	9	GSM7681306	GSM7681306	eye stalk		15
SRR25525710	102	1955999532	PRJNA1002268	SAMN36843232	9	GSM7681306	GSM7681306	eye stalk		15
SRR25525711	102	1786874964	PRJNA1002268	SAMN36843233	9	GSM7681305	GSM7681305	eye stalk		15
SRR25525712	102	1797238470	PRJNA1002268	SAMN36843233	9	GSM7681305	GSM7681305	eye stalk		15
SRR25525713	102	1734273564	PRJNA1002268	SAMN36843234	9	GSM7681304	GSM7681304	eye stalk		15
SRR25525714	102	1763632428	PRJNA1002268	SAMN36843234	9	GSM7681304	GSM7681304	eye stalk		15
SRR25525715	102	1902191880	PRJNA1002268	SAMN36843235	9	GSM7681303	GSM7681303	eye stalk		15
SRR25525716	102	1907696004	PRJNA1002268	SAMN36843235	9	GSM7681303	GSM7681303	eye stalk		15
SRR25525717	102	2123165904	PRJNA1002268	SAMN36843236	9	GSM7681302	GSM7681302	eye stalk		12
SRR25525718	102	2231727870	PRJNA1002268	SAMN36843236	9	GSM7681302	GSM7681302	eye stalk		12
SRR25525719	102	1900625058	PRJNA1002268	SAMN36843237	9	GSM7681301	GSM7681301	eye stalk		12
SRR25525720	102	2026882800	PRJNA1002268	SAMN36843237	9	GSM7681301	GSM7681301	eye stalk		12
SRR25525721	102	2211360510	PRJNA1002268	SAMN36843238	9	GSM7681300	GSM7681300	eye stalk		12
SRR25525722	102	2335984416	PRJNA1002268	SAMN36843238	9	GSM7681300	GSM7681300	eye stalk		12
SRR25525723	102	2179953282	PRJNA1002268	SAMN36843239	9	GSM7681299	GSM7681299	eye stalk		12
SRR25525724	102	2301027690	PRJNA1002268	SAMN36843239	9	GSM7681299	GSM7681299	eye stalk		12
SRR25525725	102	1970328900	PRJNA1002268	SAMN36843240	9	GSM7681298	GSM7681298	eye stalk		9
SRR25525726	102	1978957080	PRJNA1002268	SAMN36843240	9	GSM7681298	GSM7681298	eye stalk		9
SRR25525727	102	1776019920	PRJNA1002268	SAMN36843241	9	GSM7681297	GSM7681297	eye stalk		9
SRR25525728	102	1816924878	PRJNA1002268	SAMN36843241	9	GSM7681297	GSM7681297	eye stalk		9
SRR25525729	102	1774789596	PRJNA1002268	SAMN36843242	9	GSM7681296	GSM7681296	eye stalk		9
SRR25525730	102	1798448700	PRJNA1002268	SAMN36843242	9	GSM7681296	GSM7681296	eye stalk		9
SRR25525731	102	1694362494	PRJNA1002268	SAMN36843243	9	GSM7681295	GSM7681295	eye stalk		9
SRR25525732	102	1792448142	PRJNA1002268	SAMN36843243	9	GSM7681295	GSM7681295	eye stalk		9
SRR25525733	102	2076511308	PRJNA1002268	SAMN36843244	9	GSM7681294	GSM7681294	eye stalk		6
SRR25525734	102	2081928528	PRJNA1002268	SAMN36843244	9	GSM7681294	GSM7681294	eye stalk		6
SRR25525735	102	2211779526	PRJNA1002268	SAMN36843245	9	GSM7681293	GSM7681293	eye stalk		6
SRR25525736	102	2247740136	PRJNA1002268	SAMN36843245	9	GSM7681293	GSM7681293	eye stalk		6
SRR25525737	102	1764113256	PRJNA1002268	SAMN36843246	9	GSM7681292	GSM7681292	eye stalk		6
SRR25525738	102	1787126700	PRJNA1002268	SAMN36843246	9	GSM7681292	GSM7681292	eye stalk		6
SRR25525739	102	2061636750	PRJNA1002268	SAMN36843247	9	GSM7681291	GSM7681291	eye stalk		6
SRR25525740	102	2067576006	PRJNA1002268	SAMN36843247	9	GSM7681291	GSM7681291	eye stalk		6
SRR25525741	102	1936135338	PRJNA1002268	SAMN36843248	9	GSM7681290	GSM7681290	eye stalk		3
SRR25525742	102	1974116568	PRJNA1002268	SAMN36843248	9	GSM7681290	GSM7681290	eye stalk		3
SRR25525743	102	2038982142	PRJNA1002268	SAMN36843249	9	GSM7681289	GSM7681289	eye stalk		3
SRR25525744	102	2054029998	PRJNA1002268	SAMN36843249	9	GSM7681289	GSM7681289	eye stalk		3
SRR25525745	102	1712981268	PRJNA1002268	SAMN36843250	9	GSM7681288	GSM7681288	eye stalk		3
SRR25525746	102	1818390414	PRJNA1002268	SAMN36843250	9	GSM7681288	GSM7681288	eye stalk		3
SRR25525747	102	1840389162	PRJNA1002268	SAMN36843251	9	GSM7681287	GSM7681287	eye stalk		3
SRR25525748	102	1842472308	PRJNA1002268	SAMN36843251	9	GSM7681287	GSM7681287	eye stalk		3
SRR25525749	102	1534946286	PRJNA1002268	SAMN36843252	9	GSM7681286	GSM7681286	eye stalk		1
SRR25525750	102	1569860682	PRJNA1002268	SAMN36843252	9	GSM7681286	GSM7681286	eye stalk		1
SRR25525751	102	1665579114	PRJNA1002268	SAMN36843253	9	GSM7681285	GSM7681285	eye stalk		1
SRR25525752	102	1752153552	PRJNA1002268	SAMN36843253	9	GSM7681285	GSM7681285	eye stalk		1
SRR25525753	102	1855519944	PRJNA1002268	SAMN36843254	9	GSM7681284	GSM7681284	eye stalk		1
SRR25525754	102	1873287528	PRJNA1002268	SAMN36843254	9	GSM7681284	GSM7681284	eye stalk		1
SRR25525755	102	1894677948	PRJNA1002268	SAMN36843255	9	GSM7681283	GSM7681283	eye stalk		1
SRR25525756	102	1879644066	PRJNA1002268	SAMN36843255	9	GSM7681283	GSM7681283	eye stalk		1
SRR25525757	102	1911457152	PRJNA1002268	SAMN36843256	9	GSM7681282	GSM7681282	eye stalk		0
SRR25525758	102	1891462806	PRJNA1002268	SAMN36843256	9	GSM7681282	GSM7681282	eye stalk		0
SRR25525759	102	1627431114	PRJNA1002268	SAMN36843257	9	GSM7681281	GSM7681281	eye stalk		0
SRR25525760	102	1676333892	PRJNA1002268	SAMN36843257	9	GSM7681281	GSM7681281	eye stalk		0
SRR25525761	102	1882216608	PRJNA1002268	SAMN36843258	9	GSM7681280	GSM7681280	eye stalk		0
SRR25525762	102	1888419534	PRJNA1002268	SAMN36843258	9	GSM7681280	GSM7681280	eye stalk		0
SRR25525763	102	1772301816	PRJNA1002268	SAMN36843259	9	GSM7681279	GSM7681279	eye stalk		0
SRR7224656	101	995446506	PRJNA473253	SAMN09269460	2	Female Digestive Gland	Female Digestive Gland	Female Digestive Gland		Adult
SRR7224655	101	1117885271	PRJNA473253	SAMN09269461	2	Fluid	Fluid	Fluid		Adult
SRR7224654	101	1048944489	PRJNA473253	SAMN09269462	2	Foot	Foot	Foot		Adult
SRR6395540	250	6788380000	PRJNA423172	SAMN08213145	1	Pca_F	Pca_F	Foot		Adult
SRR7224653	101	2385845129	PRJNA473253	SAMN09269463	2	Ganglia	Ganglia	Ganglia		Adult
SRR6429140	284	7416149659	PRJNA427478	SAMN08242148	6	GL2	GL2	gill	biologilca replicate 2	
SRR6429141	280	1716064847	PRJNA427478	SAMN08242149	6	GL3	GL3	gill	biologilca replicate 3	
SRR6429154	286	7455855287	PRJNA427478	SAMN08242147	6	GL1	GL1	gill	biologilca replicate 1	
SRR6395541	250	6152188500	PRJNA423172	SAMN08213146	1	Pca_G	Pca_G	gill		Adult
SRR31174508	302	7291405856	PRJNA1178854	SAMN44484602	8	S_10	SFG2	gill		
SRR31174509	302	7275270600	PRJNA1178854	SAMN44484601	8	S_9	SFG1	gill		
SRR31174514	302	7536845182	PRJNA1178854	SAMN44484624	8	S_32	CMG4	gill		
SRR31174515	302	7307263876	PRJNA1178854	SAMN44484623	8	S_31	CMG3	gill		
SRR31174517	302	8413217170	PRJNA1178854	SAMN44484622	8	S_30	CMG2	gill		
SRR31174518	302	8850100336	PRJNA1178854	SAMN44484621	8	S_29	CMG1	gill		
SRR31174519	302	6291217190	PRJNA1178854	SAMN44484620	8	S_16	SMG4	gill		
SRR31174520	302	6485773140	PRJNA1178854	SAMN44484619	8	S_15	SMG3	gill		
SRR31174521	302	6651693450	PRJNA1178854	SAMN44484618	8	S_14	SMG2	gill		
SRR31174522	302	6403387540	PRJNA1178854	SAMN44484617	8	S_13	SMG1	gill		
SRR31174532	302	6264429186	PRJNA1178854	SAMN44484608	8	S_28	CFG4	gill		
SRR31174533	302	7328747250	PRJNA1178854	SAMN44484607	8	S_27	CFG3	gill		
SRR31174534	302	6771020294	PRJNA1178854	SAMN44484606	8	S_26	CFG2	gill		

SRR31174535	302	8358296054	PRJNA1178854	SAMN44484605	8	S_25	CFG1	gill		
SRR31174536	302	8218222112	PRJNA1178854	SAMN44484604	8	S_12	SFG4	gill		
SRR31174537	302	7397406648	PRJNA1178854	SAMN44484603	8	S_11	SFG3	gill		
SRR7224652	101	6387162028	PRJNA473253	SAMN09269464	2	Gills	Gills	gill		Adult
SRR26589237	302	6385066106	PRJNA1033725	SAMN38043546	10	B_5	B5	gonadal		
SRR26589238	302	6919077908	PRJNA1033725	SAMN38043545	10	B_4	B4	gonadal		
SRR26589239	302	6097590494	PRJNA1033725	SAMN38043544	10	B_3	B3	gonadal		
SRR26589240	302	6288087262	PRJNA1033725	SAMN38043543	10	B_2	B2	gonadal		
SRR26589241	302	6375412072	PRJNA1033725	SAMN38043542	10	B_1	B1	gonadal		
SRR26589242	302	6939690616	PRJNA1033725	SAMN38043541	10	A_5	A5	gonadal		
SRR26589243	302	6772560192	PRJNA1033725	SAMN38043540	10	A_4	A4	gonadal		
SRR26589244	302	6828209732	PRJNA1033725	SAMN38043539	10	A_3	A3	gonadal		
SRR26589245	302	6487338104	PRJNA1033725	SAMN38043549	10	C_3	C3	gonadal		
SRR26589246	302	6440298584	PRJNA1033725	SAMN38043548	10	C_2	C2	gonadal		
SRR26589247	302	7463255936	PRJNA1033725	SAMN38043547	10	C_1	C1	gonadal		
SRR26589248	302	6364878916	PRJNA1033725	SAMN38043538	10	A_2	A2	gonadal		
SRR26589249	302	6292402238	PRJNA1033725	SAMN38043537	10	A_1	A1	gonadal		
SRR7224651	101	2138335135	PRJNA473253	SAMN09269465	2	Gut	Gut	Gut		Adult
SRR7224650	101	2537341291	PRJNA473253	SAMN09269466	11	Heart	Heart	Heart		Adult
SRR7454785	300	6687459300	PRJNA476647	SAMN09459802	11	B1M3	B1M3	hemolymph	BACT	
SRR7454788	300	6668982000	PRJNA476647	SAMN09459803	11	B1M7	B1M7	hemolymph	BACT	
SRR7454787	300	6686281500	PRJNA476647	SAMN09459804	11	B1M18	B1M18	hemolymph	BACT	
SRR7454790	300	6644325600	PRJNA476647	SAMN09459805	11	B2M1	B2M1	hemolymph	BACT	
SRR7454789	300	6739169100	PRJNA476647	SAMN09459806	11	B2M7	B2M7	hemolymph	BACT	
SRR7454792	300	6687958800	PRJNA476647	SAMN09459807	11	B2M14	B2M14	hemolymph	BACT	
SRR7454791	300	6620497800	PRJNA476647	SAMN09459808	11	B3M2	B3M2	hemolymph	BACT	
SRR7454784	300	6669804300	PRJNA476647	SAMN09459809	11	B3M8	B3M8	hemolymph	BACT	
SRR7454783	300	6512412600	PRJNA476647	SAMN09459810	11	B3M16	B3M16	hemolymph	BACT	
SRR7454775	300	6461520900	PRJNA476647	SAMN09459781	11	KM3	KM3	hemolymph	CTRL	
SRR7454776	300	6577356600	PRJNA476647	SAMN09459782	11	A0M1	A0M1	hemolymph	CTRL	
SRR7454777	300	6607962600	PRJNA476647	SAMN09459783	11	A0M7	A0M7	hemolymph	CTRL	
SRR7454778	300	6618167100	PRJNA476647	SAMN09459784	11	X1M3	X1M3	hemolymph	DORM	
SRR7454779	300	6518100000	PRJNA476647	SAMN09459785	11	X1M11	X1M11	hemolymph	DORM	
SRR7454780	300	6378843900	PRJNA476647	SAMN09459786	11	X1M18	X1M18	hemolymph	DORM	
SRR7454781	300	6762989700	PRJNA476647	SAMN09459787	11	X2M5	X2M5	hemolymph	DORM	
SRR7454782	300	6622289400	PRJNA476647	SAMN09459788	11	X2M14	X2M14	hemolymph	DORM	
SRR7454773	300	6756290400	PRJNA476647	SAMN09459789	11	X2M21	X2M21	hemolymph	DORM	
SRR7454774	300	6732840300	PRJNA476647	SAMN09459790	11	X3M2	X3M2	hemolymph	DORM	
SRR7454817	300	6732666600	PRJNA476647	SAMN09459791	11	X3M14	X3M14	hemolymph	DORM	
SRR7454818	300	6726672900	PRJNA476647	SAMN09459792	11	X3M19	X3M19	hemolymph	DORM	
SRR7454819	300	6619336800	PRJNA476647	SAMN09459793	11	A2M2	A2M2	hemolymph	METAL	
SRR7454820	300	6502136100	PRJNA476647	SAMN09459794	11	A2M10	A2M10	hemolymph	METAL	
SRR7454813	300	6550366800	PRJNA476647	SAMN09459795	11	A2M15	A2M15	hemolymph	METAL	
SRR7454814	300	6508477500	PRJNA476647	SAMN09459796	11	A3M4	A3M4	hemolymph	METAL	
SRR7454815	300	6602401800	PRJNA476647	SAMN09459797	11	A3M13	A3M13	hemolymph	METAL	
SRR7454816	300	6501039900	PRJNA476647	SAMN09459798	11	A3M15	A3M15	hemolymph	METAL	
SRR7454811	300	6705863400	PRJNA476647	SAMN09459799	11	A4M2	A4M2	hemolymph	METAL	
SRR7454812	300	6603507600	PRJNA476647	SAMN09459800	11	A4M7	A4M7	hemolymph	METAL	
SRR7454786	300	6702351000	PRJNA476647	SAMN09459801	11	A4M17	A4M17	hemolymph	METAL	
SRR7454805	300	6521281500	PRJNA476647	SAMN09459811	11	G1M1	G1M1	hemolymph	NEMA	
SRR7454807	300	6911382000	PRJNA476647	SAMN09459812	11	G1M11	G1M11	hemolymph	NEMA	
SRR7454802	300	6603560700	PRJNA476647	SAMN09459813	11	G1M19	G1M19	hemolymph	NEMA	
SRR7454803	300	6626056500	PRJNA476647	SAMN09459814	11	G2M5	G2M5	hemolymph	NEMA	
SRR7454793	300	6552178800	PRJNA476647	SAMN09459815	11	G2M11	G2M11	hemolymph	NEMA	
SRR7454806	300	6402323700	PRJNA476647	SAMN09459816	11	G2M14	G2M14	hemolymph	NEMA	
SRR7454808	300	6524568300	PRJNA476647	SAMN09459817	11	G3M4	G3M4	hemolymph	NEMA	
SRR7454809	300	6624233400	PRJNA476647	SAMN09459818	11	G3M21	G3M21	hemolymph	NEMA	
SRR7454804	300	6576439800	PRJNA476647	SAMN09459819	11	G3M23	G3M23	hemolymph	NEMA	
SRR7454810	300	6187871100	PRJNA476647	SAMN09459820	11	N1M2	N1M2	hemolymph	PEST	
SRR7454801	300	6609121800	PRJNA476647	SAMN09459821	11	N1M12	N1M12	hemolymph	PEST	
SRR7454800	300	6601017000	PRJNA476647	SAMN09459822	11	N1M16	N1M16	hemolymph	PEST	
SRR7454799	300	6687701700	PRJNA476647	SAMN09459823	11	N2M15	N2M15	hemolymph	PEST	
SRR7454798	300	6617446500	PRJNA476647	SAMN09459824	11	N2M4	N2M4	hemolymph	PEST	
SRR7454797	300	6422133000	PRJNA476647	SAMN09459825	11	N2M7	N2M7	hemolymph	PEST	
SRR7454796	300	6590743500	PRJNA476647	SAMN09459826	11	N3M3	N3M3	hemolymph	PEST	
SRR7454795	300	6629202000	PRJNA476647	SAMN09459827	11	N3M14	N3M14	hemolymph	PEST	
SRR7454794	300	6721274100	PRJNA476647	SAMN09459828	11	N3M16	N3M16	hemolymph	PEST	
SRR6429134	300	6611167800	PRJNA427478	SAMN08242154	6	Ht2	Ht2	hemolymph	biologilca replicate 2	
SRR6429135	300	6038126100	PRJNA427478	SAMN08242155	6	Ht3	Ht3	hemolymph	biologilca replicate 3	
SRR6429136	300	6017325900	PRJNA427478	SAMN08242152	6	Hm3	Hm3	hemolymph	biologilca replicate 3	
SRR6429137	300	5681952900	PRJNA427478	SAMN08242153	6	Ht1	Ht1	hemolymph	biologilca replicate 1	
SRR6429138	300	6032653500	PRJNA427478	SAMN08242150	6	Hm1	Hm1	hemolymph	biologilca replicate 1	
SRR6429139	300	7015642500	PRJNA427478	SAMN08242151	6	Hm2	Hm2	hemolymph	biologilca replicate 2	
SRR6429149	300	6292785600	PRJNA427478	SAMN08242140	6	Cd3	Cd3	hemolymph	biologilca replicate 3	
SRR6429151	300	5922824100	PRJNA427478	SAMN08242138	6	Cd1	Cd1	hemolymph	biologilca replicate 1	
SRR6429152	300	6067339500	PRJNA427478	SAMN08242139	6	Cd2	Cd2	hemolymph	biologilca replicate 2	
SRR6429155	300	5674058100	PRJNA427478	SAMN08242165	6	Se1	Se1	hemolymph	biologilca replicate 1	
SRR6429163	300	7569424800	PRJNA427478	SAMN08242167	6	Se3	Se3	hemolymph	biologilca replicate 3	
SRR6429164	300	5866739700	PRJNA427478	SAMN08242166	6	Se2	Se2	hemolymph	biologilca replicate 2	
SRR6429156	287	6795437694	PRJNA427478	SAMN08242164	6	P3	P3	hemolymph	biologilca replicate 3	
SRR6429157	284	7363743171	PRJNA427478	SAMN08242163	6	P2	P2	hemolymph	biologilca replicate 2	
SRR6429158	288	7514022191	PRJNA427478	SAMN08242162	6	P1	P1	hemolymph	biologilca replicate 1	
SRR7224659	101	2671969140	PRJNA473253	SAMN09269467	2	hemolymphs	hemolymphs	hemolymph		

SRR6490387	300	8885775600	PRJNA429701	SAMN08357953	8	PRJNA429701	Transcriptome analysis gene expression patterns of Pomacea canaliculated hemolymphs	hemolymph		
SRR7216642	250	5774229500	PRJNA473031	SAMN09258742	1	Pomacea canaliculata:J	Pca_J	juvenile		
SRR7224657	101	526382508	PRJNA473253	SAMN09269459	2	Anterior_Kidney	Anterior_Kidney	kidney	NO!	
SRR6429132	278	7227446431	PRJNA427478	SAMN08242156	6	Kn1	Kn1	posterior kidney	biologilca replicate 1	
SRR6429133	276	7162753152	PRJNA427478	SAMN08242157	6	Kn2	Kn2	posterior kidney	biologilca replicate 2	
SRR6429162	277	2144237352	PRJNA427478	SAMN08242158	6	Kn3	Kn3	posterior kidney	biologilca replicate 3	
SRR6395542	250	6314800750	PRJNA423172	SAMN08213147	1	Pca_K	Pca_K	posterior kidney		
SRR7224682	101	1299789604	PRJNA473253	SAMN09269472	2	Posterior_Kidney	Posterior_Kidney	posterior kidney		
SRR7224658	101	665919260	PRJNA473253	SAMN09269468	2	Lung	Lung	lung		
SRR6395543	250	5110790250	PRJNA423172	SAMN08213148	1	Pca_L	Pca_L	lung		
SRR7224679	101	1352066396	PRJNA473253	SAMN09269469	2	Male Digestive Gland	Male Digestive Gland	Male Digestive Gland		
SRR7224680	101	495916464	PRJNA473253	SAMN09269470	2	Mantle	Mantle	mantle		
SRR6395544	250	6118928250	PRJNA423172	SAMN08213149	1	PCa_M	Pca_M	mantle		
SRR29790670	300	6840747300	PRJNA1134816	SAMN42438496	12	niclosamide	niclosamide	not applicable		
SRR29790672	300	7102725300	PRJNA1134816	SAMN42438495	12	mebendazole	mebendazole	not applicable		
SRR29790673	300	6906649800	PRJNA1134816	SAMN42438494	12	control	control	not applicable		
SRR7224681	101	6420325176	PRJNA473253	SAMN09269471	2	Ovaries	Ovaries	ovary		
SRR6429159	284	2749887228	PRJNA427478	SAMN08242161	6	Ov3	Ov3	ovary	biologilca replicate 3	
SRR6429160	283	7371851104	PRJNA427478	SAMN08242160	6	Ov2	Ov2	ovary	biologilca replicate 2	
SRR6429161	286	7448877741	PRJNA427478	SAMN08242159	6	Ov1	Ov1	ovary	biologilca replicate 1	
SRR17191967	294	7139784380	PRJNA787768	SAMN23843810	13	Control_3	Control_3	soft tissues		
SRR17191968	294	7028251983	PRJNA787768	SAMN23843809	13	Control_2	Control_2	soft tissues		
SRR17191969	293	7140449844	PRJNA787768	SAMN23843808	13	Control_1	Control_1	soft tissues		
SRR17191970	292	7050026869	PRJNA787768	SAMN23843807	13	PBQ_3	PBQ_3	soft tissues		
SRR17191971	294	7121449779	PRJNA787768	SAMN23843806	13	PBQ_2	PBQ_2	soft tissues		
SRR17191972	293	6525317261	PRJNA787768	SAMN23843805	13	PBQ_1	PBQ_1	soft tissues		
SRR7224683	101	172045016	PRJNA473253	SAMN09269473	2	Stomach	Stomach	stomach		Adult
SRR6395545	250	5607533000	PRJNA423172	SAMN08213150	1	Pca_S	Pca_S	stomach		Adult
SRR7224684	101	5007989656	PRJNA473253	SAMN09269474	2	Testis	Testis	testis		Adult
SRR6429142	285	7429530913	PRJNA427478	SAMN08242168	6	Te1	Te1	testis	biologilca replicate 1	
SRR6429143	282	7342321091	PRJNA427478	SAMN08242169	6	Te2	Te2	testis	biologilca replicate 2	
SRR6429144	280	1612441769	PRJNA427478	SAMN08242170	6	Te3	Te3	testis	biologilca replicate 3	
SRR6395537	250	6538022250	PRJNA423172	SAMN08213151	1	Pca_T	Pca_T	testis		Adult
SRR11610574	300	11155344600	PRJNA629998	SAMN14731702	14	FSL-GZ	FSL-GZ	total		
SRR7224629	51	1140775140	PRJNA473253	SAMN09269447	2	2d_I	2d	total		2dpf
SRR7224630	51	1337851686	PRJNA473253	SAMN09269447	2	2d_II	2d	total		2dpf
SRR7224631	51	1253993916	PRJNA473253	SAMN09269447	2	2d_III	2d	total		2dpf
SRR7224632	51	1404572487	PRJNA473253	SAMN09269448	2	3d_I	3d	total		3dpf
SRR7224633	51	1432607289	PRJNA473253	SAMN09269448	2	3d_II	3d	total		3dpf
SRR7224634	51	1384146834	PRJNA473253	SAMN09269448	2	3d_III	3d	total		3dpf
SRR7224635	51	1357529577	PRJNA473253	SAMN09269449	2	4d_I	4d	total		4dpf
SRR7224636	51	1445117946	PRJNA473253	SAMN09269449	2	4d_II	4d	total		4dpf
SRR7224637	51	1162594164	PRJNA473253	SAMN09269449	2	4d_III	4d	total		4dpf
SRR7224638	51	1439878818	PRJNA473253	SAMN09269450	2	5d_I	5d	total		5dpf
SRR7224639	101	2919746380	PRJNA473253	SAMN09269457	2	19d_III	19d	total		19dpf
SRR7224640	101	2688710900	PRJNA473253	SAMN09269453	2	9d_III	9d	total		9dpf
SRR7224641	101	1514291081	PRJNA473253	SAMN09269453	2	9d_II	9d	total		9dpf
SRR7224642	101	1611422882	PRJNA473253	SAMN09269454	2	11d_I	11d	total		11dpf
SRR7224643	101	2477101962	PRJNA473253	SAMN09269453	2	9d_IV	9d	total		9dpf
SRR7224644	101	1243963369	PRJNA473253	SAMN09269454	2	11d_III	11d	total		11dpf
SRR7224645	101	927149498	PRJNA473253	SAMN09269454	2	11d_II	11d	total		11dpf
SRR7224646	101	1725128480	PRJNA473253	SAMN09269455	2	13d_I	13d	total		13dpf
SRR7224647	101	3559575522	PRJNA473253	SAMN09269454	2	11d_IV	11d	total		11dpf
SRR7224648	101	1990284487	PRJNA473253	SAMN09269455	2	13d_III	13d	total		13dpf
SRR7224649	101	1205120890	PRJNA473253	SAMN09269455	2	13d_II	13d	total		13dpf
SRR7224660	101	1952708245	PRJNA473253	SAMN09269456	2	16d_II	16d	total		16dpf
SRR7224661	101	3159919936	PRJNA473253	SAMN09269456	2	16d_III	16d	total		16dpf
SRR7224662	101	2851228889	PRJNA473253	SAMN09269455	2	13d_IV	13d	total		13dpf
SRR7224663	101	6149467416	PRJNA473253	SAMN09269456	2	16d_I	16d	total		16dpf
SRR7224664	101	2341116370	PRJNA473253	SAMN09269457	2	19d_II	19d	total		19dpf
SRR7224665	51	1407992394	PRJNA473253	SAMN09269451	2	6d_III	6d	total		6dpf
SRR7224666	101	492739004	PRJNA473253	SAMN09269452	2	7d_I	7d	total		7dpf
SRR7224667	101	1347786521	PRJNA473253	SAMN09269452	2	7d_II	7d	total		7dpf
SRR7224668	101	1270018844	PRJNA473253	SAMN09269452	2	7d_III	7d	total		7dpf
SRR7224669	51	1100061381	PRJNA473253	SAMN09269450	2	5d_II	5d	total		5dpf
SRR7224670	51	1124692749	PRJNA473253	SAMN09269450	2	5d_III	5d	total		5dpf
SRR7224671	51	1542784017	PRJNA473253	SAMN09269451	2	6d_I	6d	total		6dpf
SRR7224672	51	1467091704	PRJNA473253	SAMN09269451	2	6d_II	6d	total		6dpf
SRR7224673	101	5609713316	PRJNA473253	SAMN09269456	2	16d_IV	16d	total		16dpf
SRR7224674	101	2155473623	PRJNA473253	SAMN09269457	2	19d_IV	19d	total		19dpf
SRR7224676	101	2395654148	PRJNA473253	SAMN09269452	2	7d_IV	7d	total		7dpf
SRR7224677	101	1829576822	PRJNA473253	SAMN09269453	2	9d_I	9d	total		9dpf
SRR7224678	101	6670334415	PRJNA473253	SAMN09269457	2	19d_I	19d	total		19dpf
SRR125493	180	2315116980	PRJNA80001	SAMN00222068	15	Pomacea canaliculata	Apple snail	total		
SRR7224685	101	1201949389	PRJNA473253	SAMN09269475	2	Vessels	Vessels	Vessels		Adult
SRR6490388	300	9028041600	PRJNA429697	SAMN08357950	8	PRJNA429697	Immune related genes expression in juveniles of Pomacea canaliculata under heat stess	Whole juvenile		
SRR7224686	101	1057389301	PRJNA473253	SAMN09269476	2	Yolk_gland	Yolk_gland	Yolk_gland		Adult

Table S2. List of the primers used for synthesis of FISH probes and qPCR reactions

FISH probe	***Pc-AIF1 _ F***	5' -GCTACTGGCGCAAAGCCTAA -3'
	Pc-AIF1 _ R	5' -ACAGTTGGGGGATCTGTCCA-3'
	SP6	5' -ATTTAGGTGACACTATAGAATACTC -3'
	T7	5' -TAATACGACTCACTATAGGG -3'
qPCR	***Pc-AIF1 _ F***	5' -GCTACTGGCGCAAAGCCTAA -3'
	Pc-AIF1 _ R	5' -TTGTGGCAGTTCCTCATCAC -3'
	Pc-RpL5 _ F	5' -CGTATGCCAGAATTGAGGGT-3'
	Pc-RpL5 _ R	5' -CAACATCCAAGTATGCACGG- 3'

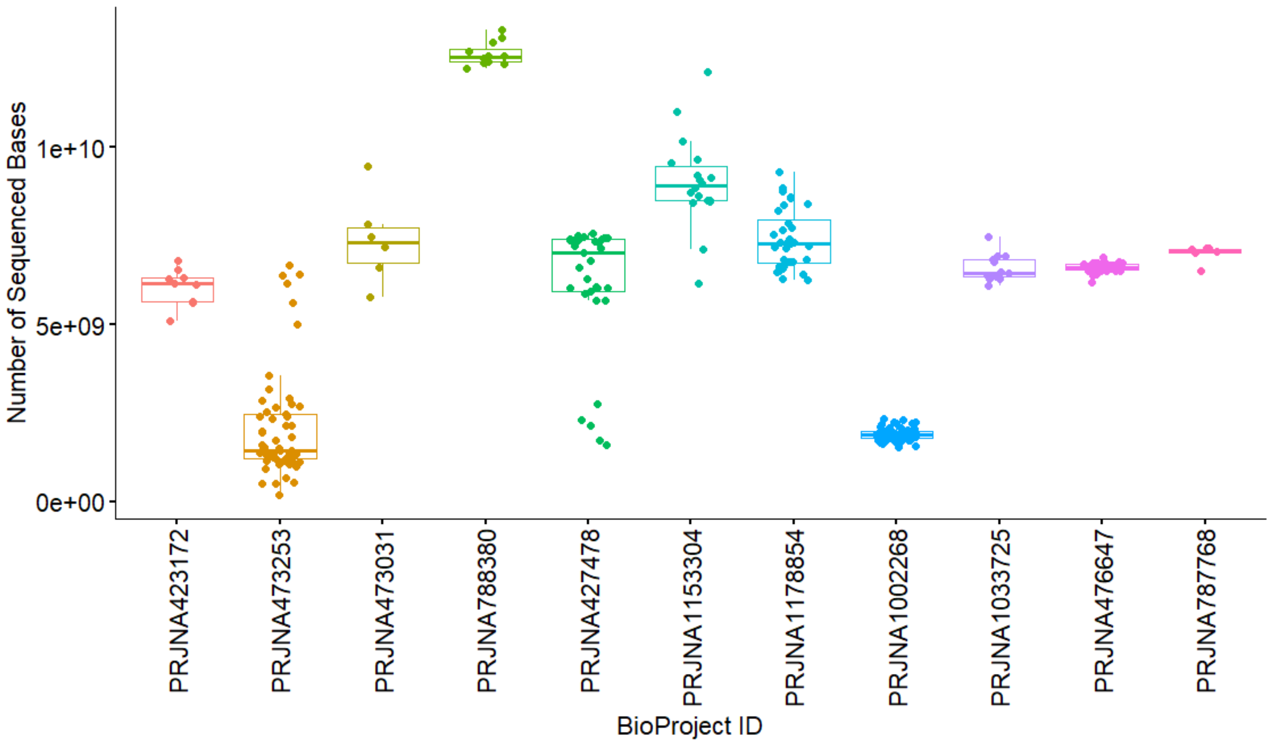

Figure S1 Sequencing depth. The number of sequenced bases is plotted with the data grouped by experiment. Only experiments with at least five samples have been considered (see Table S1 for further details).

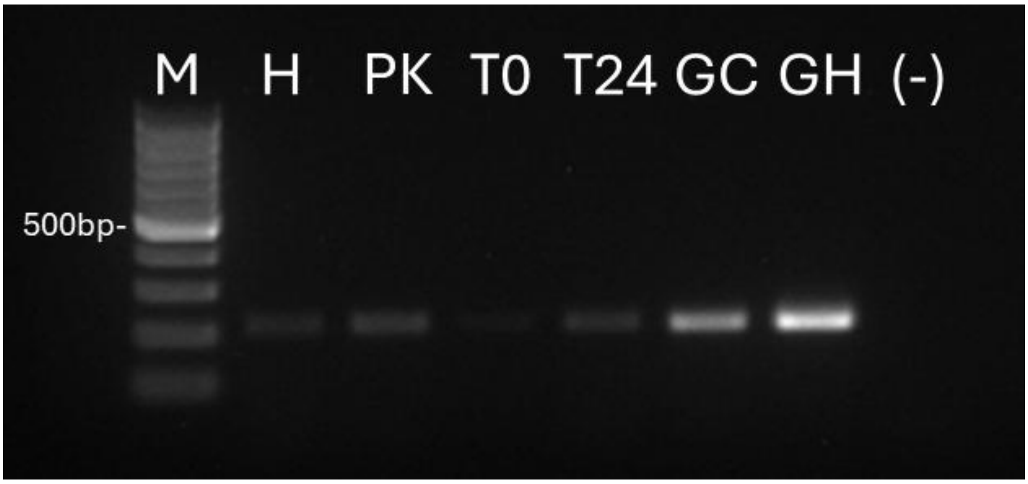

Figure S2 RT-PCR on cDNA from target organs of *P. canaliculata* performed with *Pc*-AIF1 primers. H = Hemocytes; PK = Posterior Kidney; T0 = left tentacles control; T24 = left tentacle 24 hpa; GC = contralateral ganglion (right); GH = ipsilateral ganglion (left); (-) = negative control (template omitted); M = DNA standard ladder 100 bp

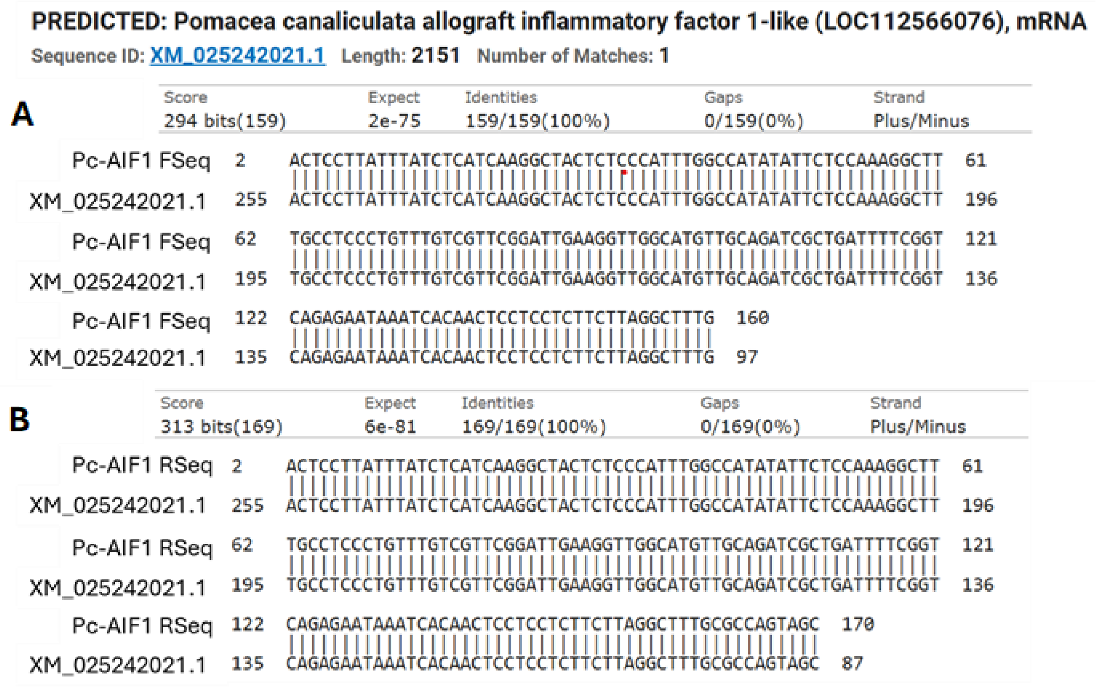

Figure S3 Nucleotide sequences of A) sequenced cDNA from left ganglion with primer *Pc*-AIF1 forward B) sequenced cDNA from right ganglion with primer *Pc*-AIF1 reverse. Lines are named according to the resulted sequence names' or with the accession number of corresponding sequence. Vertical bars between aligned nucleotides indicate a match between sequences.

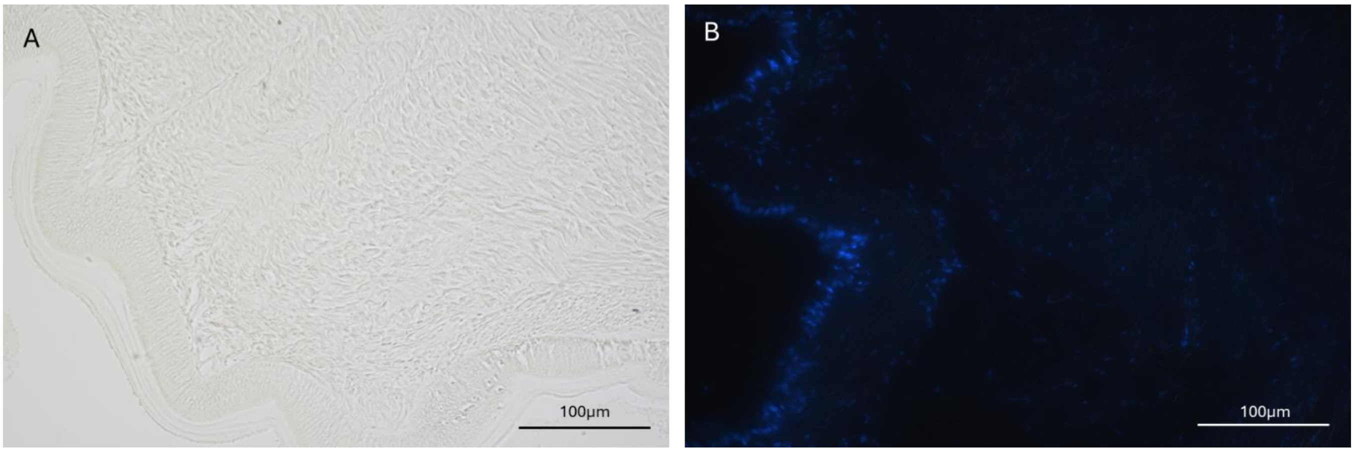

Figure S4 FISH and immunohistochemical reaction on *P. canaliculata* stomach. A) Immunohistochemical reaction with RCA120 in a tissue without hemocytes (stomach) (40× objective); B) FISH performed with *Pc*-AIF1 probe on stomach section (40× objective). Scale bars= 100 µm

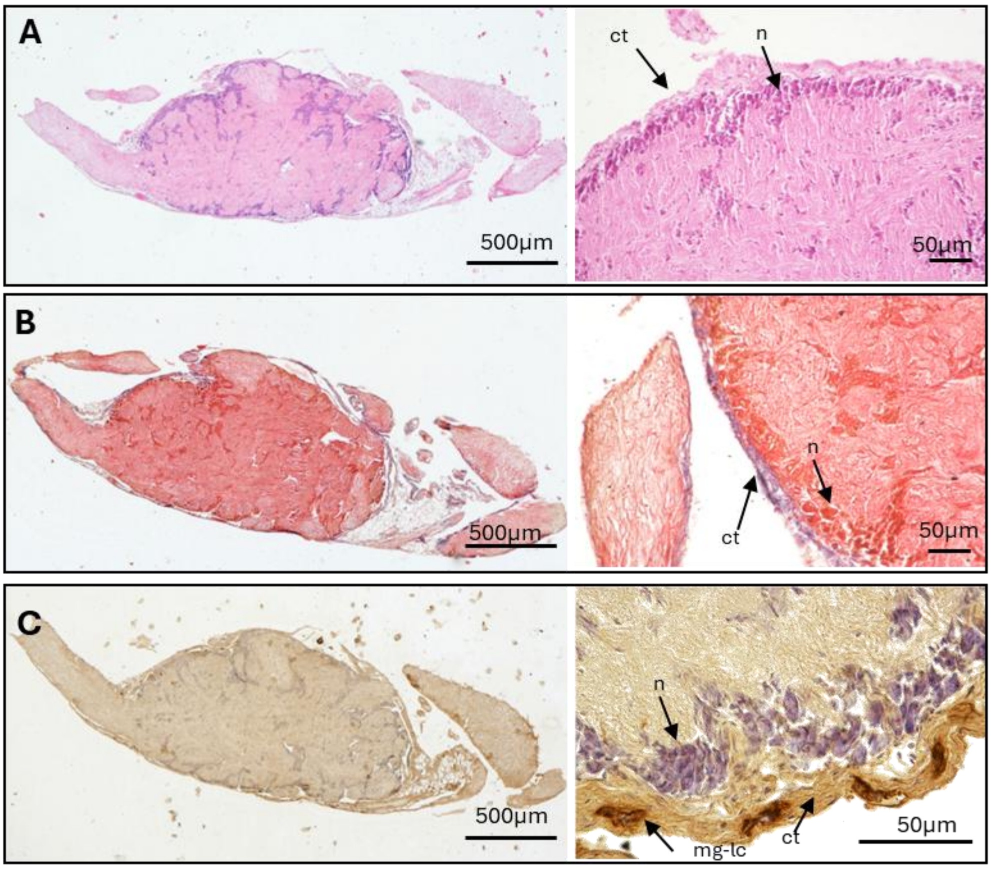

Figure S5. Histological analysis of a cerebral ganglion components stained with A) Hematoxylin-Eosin and B) Masson's Trichrome (the connective tissue is in blue); C) Identification of microglia-like cells in a cerebral ganglion using RCA120 lectin staining. RCA120-positive cells at low magnification (left) and a detailed view, highlighting their localization (right). RCA120-positive microglia-like cells are visible in the connective tissue adjacent to the ganglion. ct=connective tissue, mg-cl=microglia-like cells; n= neurons.

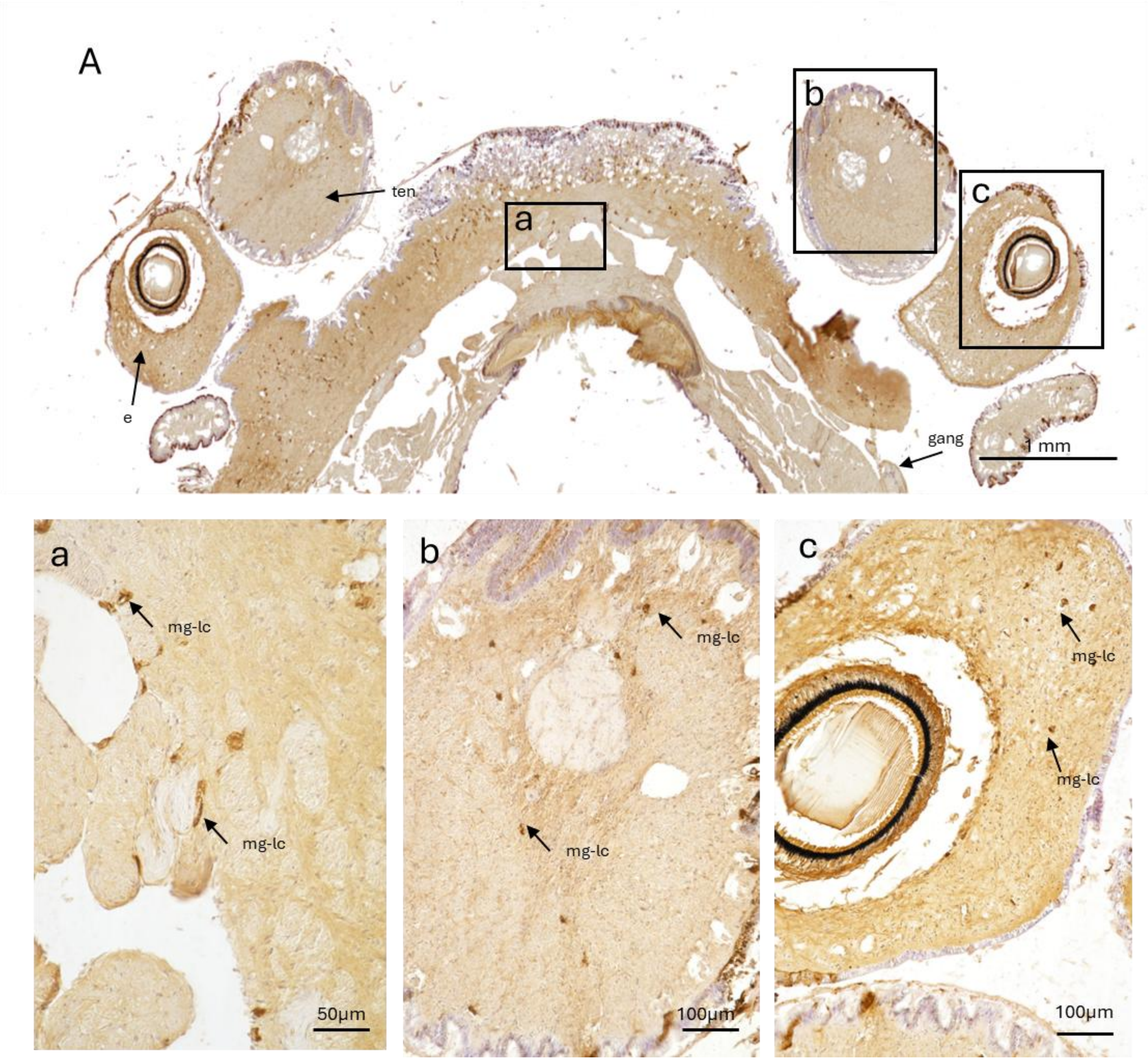

Figure S6. Identification of microglial cells in transversal head section using RCA120 lectin staining. A) Image tiling of the entire *Pomacea* head. RCA120-positive microglial-like cells are visible in the connective tissue adjacent to the nervous system. Insets: a) neural cordon connecting left and right cerebral ganglia, b) cephalic tentacle and c) eye. e=eye; mg-lc=microglia-like cell; ten= tentacle; gang=ganglia.

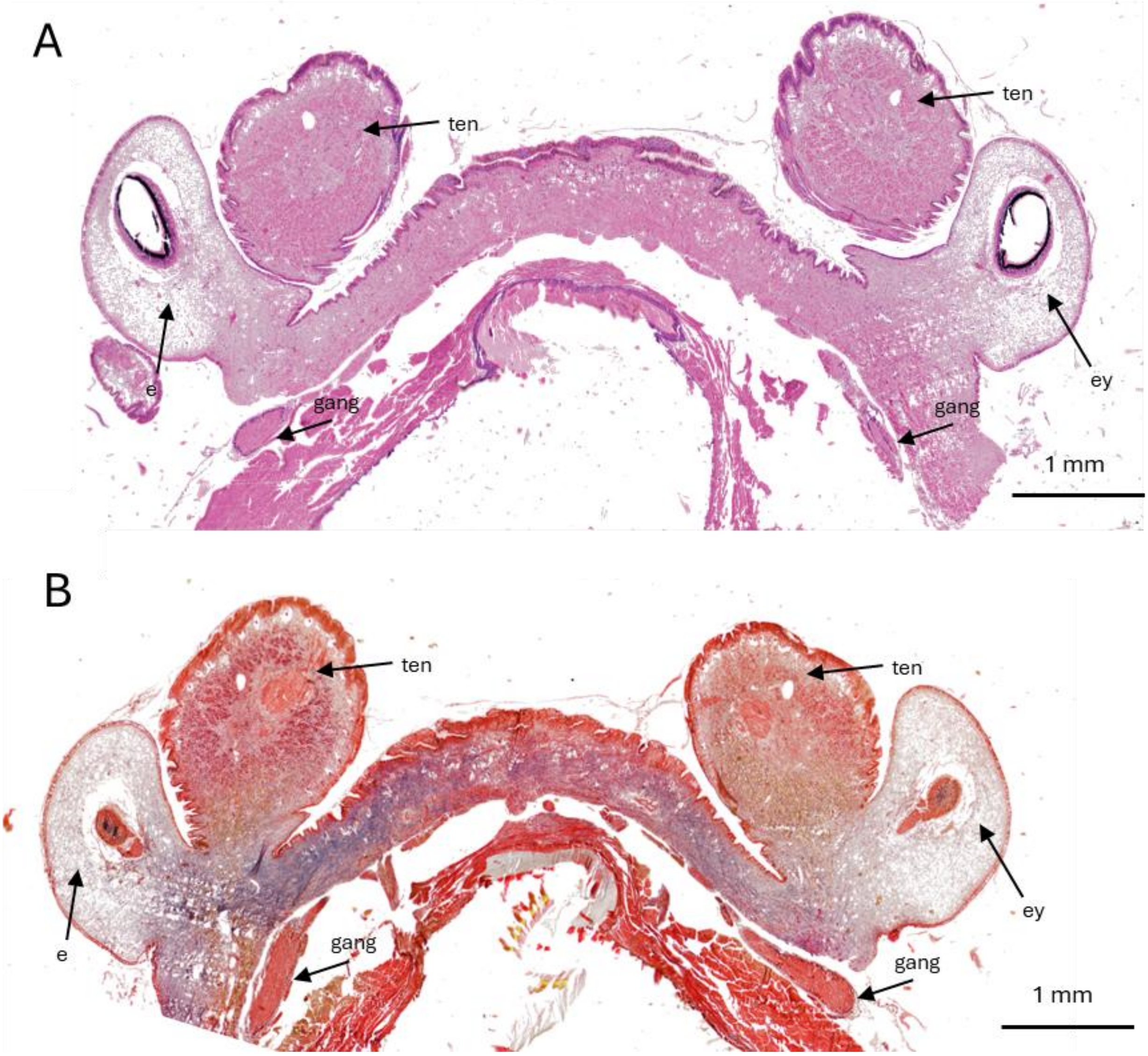

Figure S7. Histological representation by tiling of a transversal section of *P. canaliculata* head after staining with: A) Hematoxylin-Eosin; B) Masson's Trichrome (the connective tissue is in blue). Abbreviations as Figure S6.

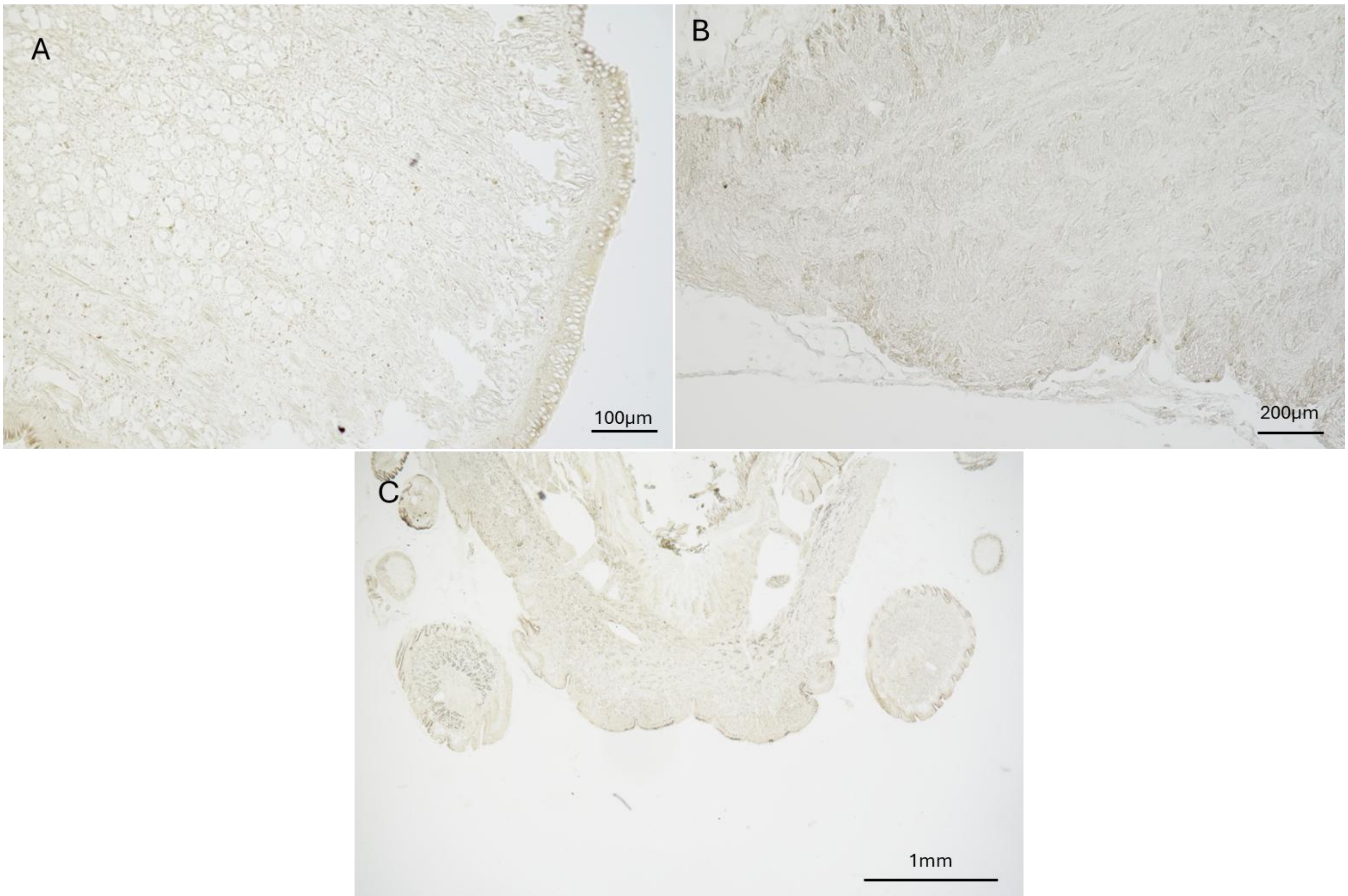

Figure S8. Negative controls of RCA120-immunostained tissues omitting the lectin. A) Cephalic tentacle 24 hpa (20× objective); B) Ganglia from a control snail (10× objective); C) Frontal cut of a whole snail head (4× objective). Scale bars= 100 µm, 200 µm, 1mm
